# Mirror neurons are modulated by grip force and reward expectation in the sensorimotor cortices (S1, M1, PMd, PMv)

**DOI:** 10.1038/s41598-021-95536-z

**Published:** 2021-08-05

**Authors:** Md Moin Uddin Atique, Joseph Thachil Francis

**Affiliations:** 1 Department of Biomedical Engineering, Cullen College of Engineering, The University of Houston, Houston, TX 77204 USA; 2 Department of Electrical and Computer Engineering, Cullen College of Engineering, The University of Houston, Houston, TX 77204 USA

**Keywords:** Attention, Motivation, Emotion, Motivation, Motor control, Reward, Sensorimotor processing, Somatosensory system

## Abstract

Mirror Neurons (MNs) respond similarly when primates make or observe grasping movements. Recent work indicates that reward expectation influences rostral M1 (rM1) during manual, observational, and Brain Machine Interface (BMI) reaching movements. Previous work showed MNs are modulated by subjective value. Here we expand on the above work utilizing two non-human primates (NHPs), one male *Macaca* Radiata (NHP S) and one female *Macaca* Mulatta (NHP P), that were trained to perform a cued reward level isometric grip-force task, where the NHPs had to apply visually cued grip-force to move and transport a virtual object. We found a population of (S1 area 1–2, rM1, PMd, PMv) units that significantly represented grip-force during manual and observational trials. We found the neural representation of visually cued force was similar during observational trials and manual trials for the same units; however, the representation was weaker during observational trials. Comparing changes in neural time lags between manual and observational tasks indicated that a subpopulation fit the standard MN definition of observational neural activity lagging the visual information. Neural activity in (S1 areas 1–2, rM1, PMd, PMv) significantly represented force and reward expectation. In summary, we present results indicating that sensorimotor cortices have MNs for visually cued force and value.

## Introduction

**Figure 1:**
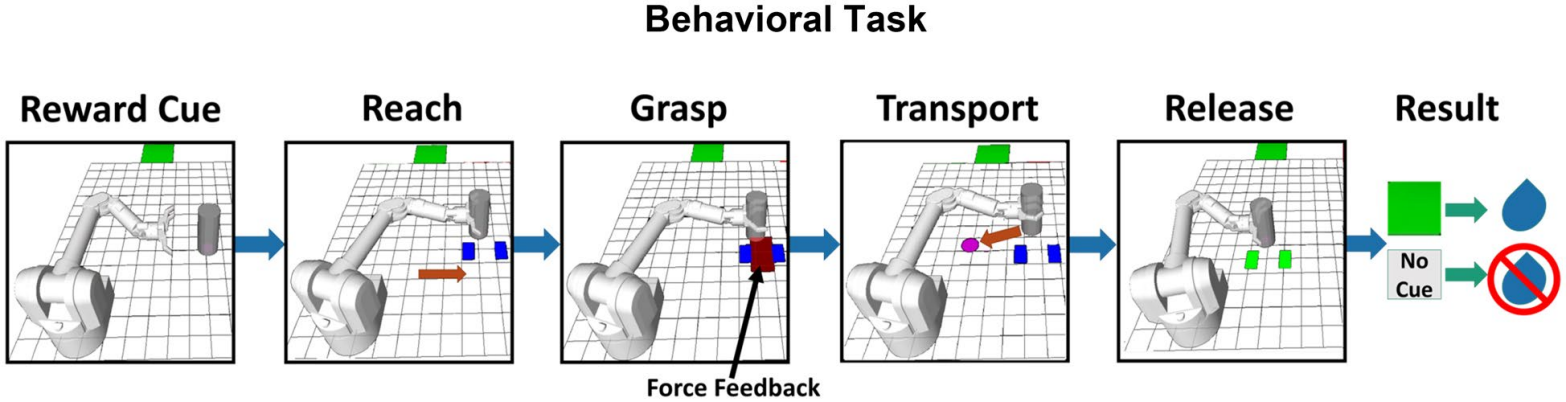
Flow diagram illustrating a single trial. The task is visually identical for both the manual and observational versions. The reward cue (green square, R1) was shown for R1 trials in cued blocks only. The absence of the green square indicated a non-rewarding (R0) trial during cued blocks of the task. The robotic arm started in a resting position for all trials (left-most image). There was no cue provided during the “Reward cue” period for any trials during uncued blocks. Please note, under experimental conditions with NHPs, the background of the grid and free space around the robotic arm and object were black. For the reader’s benefit and visual clarity, both areas have been changed to white here. Blue rectangles are grip-force targets and were seen for the duration of a trial. The red rectangle shows visual force feedback of the NHPs grip-force output initially but is no longer shown once the NHP has reached the acceptable force range (blue rectangles).

Mirror neuron (MN) activity has been observed while studying kinematic behaviors^1–4^ and through indirect measures in human motor cortex^5^. In order to ask questions about force information at the single-unit level, we utilized an isometric grip-force paradigm (Fig. 1) and cued reward level^6,7^ while modulating both cued grip-force and reward level in the current work. We utilized this grip-force paradigm initially for brain machine interfacing research^8^. Subsequently, we included observation-only trials to ask questions about MN activity within the sensorimotor regions when kinematics are not actively controlled, but rather force output is the controlled variable.

A better understanding of how reward expectation influences the primary- and pre- sensorimotor cortices has practical implications for producing stable and autonomously updating brain-machine interfaces (BMIs)^6,7,9–12^. Neural modulation related to reward has been well characterized within many brain structures^13–15^, and is known to occur at many levels, such as single units and local field potentials (LFP) within rM1^6^. The neural response to both reward and conditioned stimuli (CS) that predict reward has been demonstrated in many brain regions^7,16–22^. It has recently been shown that reward expectation changes directional and force-related tuning functions within the primary sensorimotor cortices during reaching movements and BMI reaching movements^8,23^. The above studies indicate the need to conduct more research on the influence reward has on these brain regions and how such variables may modulate MNs. The influence of reward on the caudal primary somatosensory cortex (cS1, area1 and 2) was reported in conference proceedings^24–26^. While these experiments demonstrated our ability to classify reward expectation from S1, they did not provide insight on its influence of grip-force during action and action observation as we do here. Although a similar reward signal has been demonstrated in primary somatosensory cortex utilizing fMRI, where a more robust BOLD response was recorded for higher reward delivery^27^, the investigation of reward-correlated signals within S1 at the single-unit level has not been fully developed. However, some examples do exist^24–26,28^.

Our previous work focused on identifying significant units related to reward and force within single blocks of data^8^ during manual trials and BMI control. We thought it important to investigate the cause behind each unit’s modulated activity across multiple blocks during both manual^8^ and observational tasks to expand on these findings. We hypothesized modulated responses during observation might be explained by the presence of mirror neural activity when NHPs observed the task, received a reward, or possibly both. To this end, our goal was to detect units that encoded the following: 1) Motor actions that are the physical application of force; 2) Cued “force” during visual observation; 3) units that encoded both 1 and 2. 4) Determine which of these (1–3) were further modulated by reward level, cued or uncued. We focused on “grasping” movements with cued isometric grip-force control to expand beyond our previous work on reaching movements^6,7^. Below we demonstrate our ability to decode actual and observed grip-force neural responses during manual and observational trials and reward’s influence on the neural population during reward cued and uncued trials. Others have described the activity of MNs in rM1 during observation of movement^3,4,7,29,30^. However, little has been reported on the activity in S1 during observational tasks or any of the 4 regions as it pertains to isometric grip-force control while modulating reward level as we do here. Some studies on humans and NHPs showed MN activation^31–33^, and grip-force related modulation^34,35^ is present in somatosensory areas, which implies the possibility of MN-related activity of grip-force in the cS1 cortex. However, recent work found little to no response in S1 in humans utilizing fMRI induced by observation of touch, indicating a lack of consensus on S1’s role during observation. Generally, our paper presents findings of MN responses related to varying levels of grip-force (Fig. 6) and reward’s influence on grip-force MN activity (Figs. 8, 9, 10) within cS1, rM1, PMd and PMv.

## Methods

All NHP manipulations described in this work were approved by the Institutional Animal Care and Use Committee of the State University of New York at Downstate Medical Center and conformed to the National Institutes of Health (NIH) and United States Department of Agriculture (USDA) animal care and use guidelines. In addition, this work complies with the ARRIVE guidelines. Two non-human primates (NHPs), one 9.0 kg male *Macaca* Radiata (NHP S) and one 5.0 kg female *Macaca* Mulatta (NHP P), were trained to perform a behavioral grip-force task using their right hand and subsequently implanted with 96-channel electrode arrays in cS1, rM1, PMd and PMv.

We describe the sequence for one complete trial of the manual reward-cued version of the task and state differences with the observational versions, which are visually identical (Fig. 1). First, the virtual robot moved to the start position autonomously, always to the far left of the task space. Second, cue scene, a green square moved across the top of the screen for 0.5 s from the left, indicating a rewarding (R1) trial, the absence of this green square indicated a non-rewarding (R0) trial during the cued version of the task. Third, the virtual robot autonomously moved its arm toward the cylindrical object in the reach scene. Fourth, during the grasping scene of manual trials, the NHPs had to apply isometric grip force using a stationary handle that contained a force transducer. The amount of force required was indicated by two blue rectangles, where the inner vertical edge of each blue rectangle indicated minimum force, and the outer edges indicated maximum force. The minimum threshold of force for NHP S was randomly chosen from either 150 or 200, and for NHP P, it was 100 or 150. If the force output went below that minimum value, the trial was considered a failure. The upper threshold was determined by randomly adding either 300 or 500 to the minimum for NHP S and adding randomly either 250 or 350 to the minimum for NHP P. Therefore, the ranges used for NHP S were randomly selected from the following, 150–450, 150–650, 200–500, 200–700, and for NHP P the ranges were, 100–350, 100–450, 150–400, 150–500. If the output force of the NHP was above these max levels, the trial was considered a failure. The peak grip force values across the manual trials, R1 and R0, in cued and uncued blocks are provided in supplementary figure S7. When the NHP’s applied force was within the acceptable tolerance range, the red force rectangle’s edges would also be within the blue rectangles. When this occurred, the visual force feedback (red rectangle) was removed, and the robot hand would grasp the object. We utilized this method so the NHPs could not simply use visual feedback to perform the manual task. Instead, they had to learn to control their force output based on the visual force targets and somatosensory feedback for manual trials. Thus, they had to learn to produce a given grip-force, and this may have led to positive results during observational tasks, as they most likely had built an internal representation of the force cues and their production of such force and the expected somatosensory feedback. During observational trials, the above information for the grasping scene was shown visually while the task program produced “force” profiles trapezoidal in speed. During these task produced “force” profiles, all of the visual information to the NHP was as in the manual version. See Figs. S6–S7 for the average force, duration, and peak force of these “force” trajectories and the manual versions. During the transport scene, the robot arm autonomously moved the object to a predetermined target location, indicated by a pink circle. If the NHPs applied the appropriate grip-force during object grasp and transport, and then released when the object was touching the virtual ground at the pink target location, the trial was successful. Feedback was provided to NHPs for successful placement when the blue force squares turned green and the robotic arm released the cylinder during the release scene. If they did not apply proper force during the initial grasp, failed to maintain an acceptable force range at any time during the grasp or transport scenes, or released their grip early or late, the trial was considered a failure.

There were four types of task blocks, which were experienced by each NHP in the following order: First, the manually performed task with the presence of a reward level conditioned stimulus (CS) (manual cued); Second, the manually performed task without a reward level CS (manual uncued); Third, the observational task with the presence of a reward level CS (observational cued); Fourth, the observational task without a CS (observational uncued). All trials (R0 and R1) during uncued blocks lacked a visual reward cue, so the NHP had no indication of trial value until the post result period, and thus, no explicitly cued expectation for reward outcome as the trial sequence was randomized with no clear autocorrelation (see supplementary Fig. S1). The time limit to complete a successful trial was within 10 s. The NHPs had to repeat failed trials under the same reward conditions until successful, thus incentivizing non-rewarding trials. Without this added stipulation, the NHPs would choose to fail R0 trials to move onto a possible R1 trial, indicating the NHPs clearly understood the cue reward values. Juice rewards were delivered via a system-controlled solenoid driven by task logic (Crist instruments). All elements of the grip-force task were developed in Linux using robot operating system (ROS)^36^. ROS and Python controlled the task logic, outputs to the reward delivery system, and provided timestamp synchronization with external systems to simultaneously and accurately record task state, reward delivery, and neural data.

During observational blocks, NHPs were visually monitored by researchers in real-time via cameras during all sessions to make sure NHPs remained attentive and focused on the projection screen, especially during observational trials. During observational trials, NHPs did not have access to the force transducer handle, and their arms were blocked behind a plexiglass box meant to keep the NHPs’ hands away from the trainers (BKIN’s Arms-Free restraint chair). Additionally, NHPs were trained and performed all experiments in a dark, quiet, and distraction-free isolation chamber to encourage their attention remained on the large projection screen. Each NHPs’ rear-mounted cranial head post was affixed to the BKIN primate chair to restrict head movement for neural recordings. The virtual environment was projected onto a vertical screen in the animal’s visual field. The visual projection of the virtual robotic system was approximately the same size as the real WAM robotic arm ~ 1 m reach (WAM Barrett). Recording sessions were broken into blocks of trials that averaged ten minutes of randomized R1 and R0 trials.

### Chronic implantation

**Figure 2:**
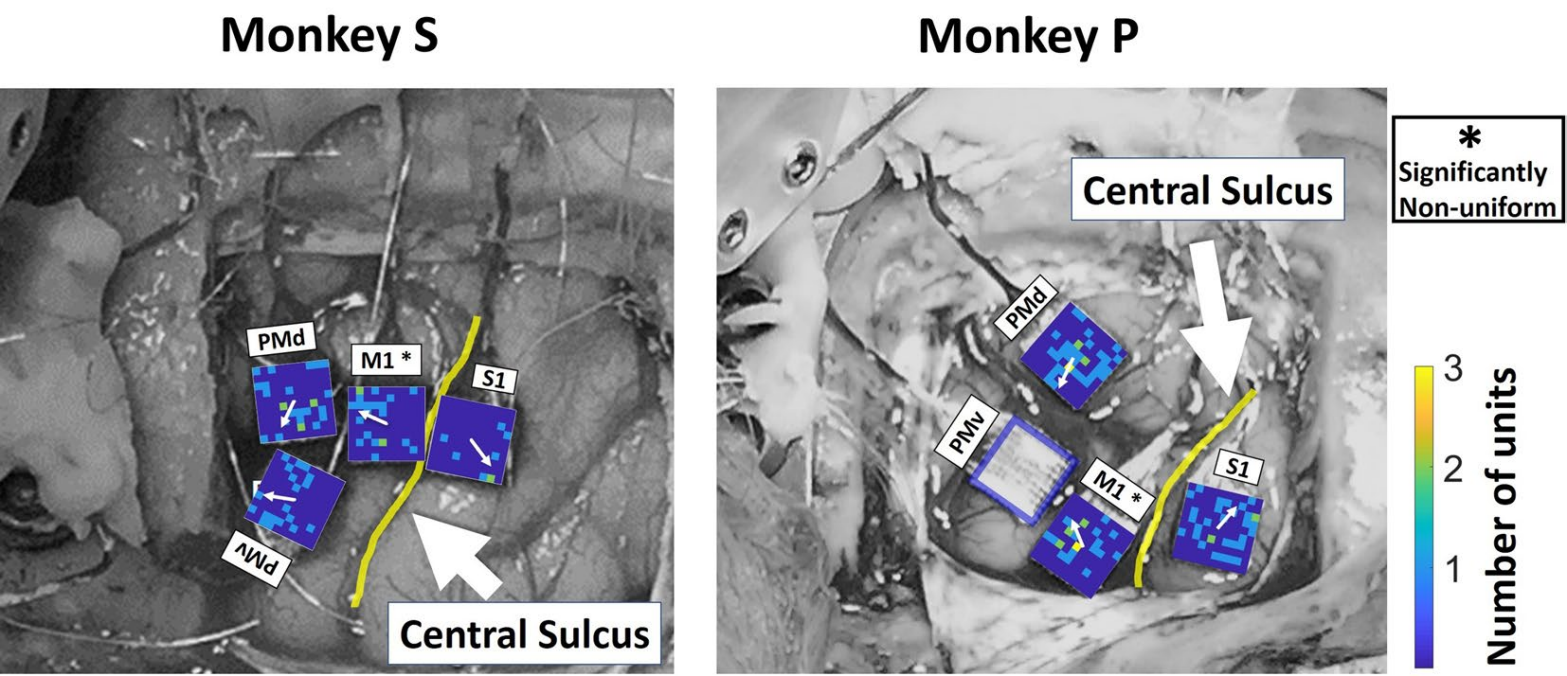
Position of four Utah arrays in relation to the central sulcus for NHP S (left) and P (right). The four arrays were implanted in cS1, rM1, PMd and PMv cortices. The yellow line indicates the Central Sulcus. Note that in NHP P, we had to implant the rM1 array more lateral than in NHP S due to a large set of blood vessels running through that region. Likewise, PMd was implanted more medial in NHP P for this exact reason than NHP S. The color map on each array is for the number of MN (mirror neuron) units recorded in each location. We did not have the electrode map for NHP P PMv. The white arrow inside the color map represents the mean direction of MN positions with respect to the array's center. A Rayleigh test (p < 0.01) showed that the distribution of MNs around the center of the array is non-uniform only for M1 in both NHPs.

We performed implantation procedures as described in-depth in a previous methods paper^37^, but give a summary here. Following training, when both NHPs achieved greater than 80% success rate on all trials in a block, the animals were implanted with chronic electrode arrays consisting of a 10 by 10 array of 1.5 mm electrodes, of which 96 were active for all regions (rM1, PMd, PMv) except cS1, which was implanted with electrodes of 1 mm length. Spacing between individual electrodes within the arrays was 400 μm (Utah array, Blackrock Microsystems) (Fig. 2). All surgical procedures were conducted using aseptic technique. NHPs were initially anesthetized with Ketamine, followed by isoflurane and a continuous infusion of fentanyl. The NHP was then placed into a stereotactic frame before the surgical site was shaved and cleaned. An incision was made along the skull to expose desired implant locations. The craniotomy window was large enough to accommodate implant locations while leaving enough margin between the dural flap and skull. The dural flap was kept under tension using stay sutures until electrode arrays were implanted and the site was ready to close. We performed intraoperative probing of cS1 to ensure implantation within the hand region using a four-shank, 32-channel silicon microelectrode array^38^ within the post-central gyrus as determined by stereotactic coordinates. The NHP’s contralateral hand was continuously stimulated by touch to assess cS1 hand region boundaries. Neural responses were amplified and sent to an audio speaker to verify stimulation areas. Utah arrays were then chronically implanted in cS1’s hand region, in rM1 directly reflected across the central sulcus rostral from cS1 for NHP S. However, NHP P had large blood vessels that interfered, and we had to implant rM1 more lateral than in NHP S as seen in Fig. 2. In addition, as seen in Fig. 2, NHP P’s PMd implant was slightly more medial than NHP P, and NHP.

S’s PMv was slightly more lateral than NHP P. In both NHPs our PMv arrays had fewer clear single units than the other arrays. We cannot be sure but believe this was due to the PMv wire bundles exiting the craniotomy closer to the array and at a stiffer region of the wire than the other arrays. The craniotomy was replaced according to previously described methods^37^.

### Neural recording

Neural recordings were performed using three synchronized multi-channel acquisition processors (Plexon, Dallas, TX), each having 128-spike waveform recording channels and 32 analog channels to record local field potentials simultaneously^6,12^. Single unit recordings were amplified and retained using waveform voltage thresholds. Thresholds were set using an auto-scale (Plexon recording software) feature, followed by manual adjustments that eliminated noise on channels before recording. Robot operating system^36^ and Python programs controlled task logic and embedded timestamps into neural recordings using a common clock. The common clock was maintained by a microprocessor that delivered a 2 kHz pulse to keep task logic and neural data synchronized. Initially, unit waveforms were automatically clustered using a k-means algorithm in principal component space^39,40^. Afterward, we used Plexon’s offline sorter software to adjust clusters to remove noise and artifacts.

### Significance related to reward

We only considered successfully completed trials for all analyses and results presented in this work. We analyzed single-unit activity from four types of task blocks for each NHP, cued or uncued, and these were either manual or observational. For trials that contained a visual reward cue, the post-cue (0–500 ms) analysis window began immediately after the green visual cue came to rest. In uncued trials, the “post-cue” period (0–500 ms) began when the robotic arm returned to its rest position from the previous trial. We defined the “post-result” period (0–500 ms) as the time after the cylindrical object was successfully placed at the target location. We analyzed spike activity in the post-cue and post-result periods separately for cued and uncued blocks to identify units significant for reward modulation. First, we binned the spike activity into non-overlapping bins of 100 ms covering 500 ms for both the post-cue and post-result periods. Next, we collected the p-values from t-test for Spearman Rank correlation, and the test was done between the binned spike rate and corresponding reward levels (0 for R0 and 1 for R1). For reward levels, 0 is used for R0, and 1 is for R1. We then extracted significant units (t-test, p < 0.05) for reward expectation (post-cue activity, R1 vs. R0 trials) and reward result (post-result activity, R1 (after reward delivery) vs. R0 (after a successful trial with no reward)). The p-values from the test are adjusted for multiple comparisons using the false discovery rate (FDR) procedure by Benjamini and Hochberg (BH Method)^41^ for the number of times the t-test was applied, which is equal to the number of units for each case, which is given in supplementary (Fig. S2). Finally, we compared spike rate activity between cued and uncued blocks to gain better insight into how reward feedback information was affected by the presence or absence of a reward cue that is reward expectation based on explicit environmental cues. Our interest in reward expectation stems from our work towards autonomously updating BMIs, thus our use of this paradigm.

### Grip-force trajectory prediction

We applied linear regression in two steps to identify units that showed a significant prediction of grip-force from cS1, rM1, PMd and PMv. In these two steps, the first step identified all units that were significantly related to grip-force, which was used below in the sections labeled “*Grip-force Tuning Curve Analysis”* and “*Identifying Observation Modulated Neurons*”. The second step was applied to sort out a selective number of units from the units collected in the first step for force prediction to reduce the number of predictors and avoid computational complexity. Unit activity from the cortices mentioned above were analyzed along with force profiles for both NHPs during manual and observation task blocks. See supplementary material for data not shown in the main text. During some trials, initially, the NHP’s applied more grip-force than necessary, and to meet the target value, they reduced their grip-force drastically. This resulted in an overcorrection where the NHP had to apply more force to meet the requirement and resulted in force profiles with multiple peaks. We manually inspected and pruned all force profiles that contained multiple peaks due to highly variable or corrective grip-forces applied by NHPs. We defined force onset as the point where force values increased from zero to a positive value of 50 au and force offset when values returned below 50 au, as the NHPs only grasped the force transducer during the grasp scene. A trial was considered successful when their output force < 50 au, which generally meant they let go of the handle. Force data were collected from 0.5 s before force onset through 0.5 s after force offset for all trials. The collected force data were smoothed using a Gaussian kernel (100 ms wide). During observational trials, the visually cued “force” targets and “force” output profiles were trapezoidal and inferred from the hand's trapezoidal velocity grasping motion. Therefore, smoothening gave the “force” profile a gaussian shape like the force data on manual blocks. We evaluated neural spike activity to the force data collected in each trial. We evaluated neural spike activity 0.5 s before and following each force sample value. This neural activity was further placed into ten non-overlapping time bins centered on each force value (100 ms bins, covering 0.5 s pre- and 0.5 s post-force sample value) to determine a unit’s significance to grip-force, so for each unit, there were 10-time bins used as predictors. We performed this separately on each unit from all four cortices. As we smoothed the unit raw data using a Gaussian kernel (100 ms wide), we wanted to find a generalized kernel width that would be used for both cued and uncued data blocks of the same kind manual vs. observational. The force prediction accuracy for different kernel bin widths from 10 to 250 ms were determined for all data. We manually selected the above value (100 ms) for the kernel width so that the predictions were close to the peak prediction values for all blocks. See Fig. S3 for differences between analysis on the smoothed data vs. unsmoothed. The collected binned data were square-root transformed to achieve a more normalized distribution. A comparison of grip-force prediction accuracy was given before and after the square-root transformation was applied (supplementary Fig. S4) to determine if the transformation affected the prediction results significantly.

Data recorded from each NHP was split into a fit (90%) and test (10%) set to check the goodness of fit and verify the predictive performance of the final linear regression model (Eq. 2). However, before testing the final linear regression model, we extracted significant units related to force in a two-step process. To do this, we subdivided the 90% fit set into 80% (fit) and 10% (validate) data subsets. See the supplementary section for a cartoon of this procedure Fig. S5.

1 $${{\varvec{y}}}_{{\varvec{f}}}={\varvec{a}}{\mathbf{X}}_{fr}^{{n}_{i}}+b$$

Equation 1 shows the linear model used, where ${{\varvec{y}}}_{f}$ is the vector of force values, ${{\varvec{X}}}_{fr}^{{n}_{i}}$ is the binned spike rate matrix for neuron ${n}_{i}$, while ${\varvec{a}}$ and $b$ are coefficients fit to the data. In the first step, we applied linear regression using Eq. 1 with the 80% subset data for the fit. We collected the F-statistic from the analysis of variance (ANOVA) for the model. Units were sorted according to their p-value from the above F-test, and those with a significant fit (p < 0.05) were considered in the second step of the process. For the second step, we took significant force units, starting with the most significant (lowest p-value), and used it to test the prediction of force values in the remaining 10% validation subset. We calculated the R-squared value after each unit was added to the model, and if the R-squared value increased (improved prediction results), the unit was kept. The remaining units that did not improve the model’s force prediction were pruned. After the two-step process, the subset of significant force units was utilized in the final linear regression model (Eq. 2). This subset of units was used with updated coefficients fit to the original 90% validation dataset. Finally, we used the held out 10% test set to determine prediction and validate the accuracy of the linear regression model (Eq. 2) by comparing them against actual recorded force values.

2 $${y}_{f}= \alpha {\mathrm{X}}_{fr}+\beta$$

The regression model used for final grip-force prediction from the neural activity is shown in Eq. 2. Here, ${y}_{f}$ denotes grip-force and, ${\mathrm{X}}_{fr}$ represents binned firing rates for the population of units being used, $\alpha$ and $\beta$ are model coefficients fit to the data.

### Grip-force tuning curve analysis

Our previous research concerning force tuning curves in rM1 showed a significant difference between R1 and R0 trials^8^. We applied the same analysis to our cS1, rM1, PMd, and PMv data to determine if neural activity led to different force tuning curves when taken from R1 vs. R0 trials. As in Zhao et al. 2018, we utilized analysis of covariance (ANCOVA). We analyzed each unit previously identified as significant for force to measure whether the slopes of the tuning curves between R1 and R0 were significantly different (F-test, p < 0.01). The p-values from the ANCOVA test are adjusted for multiple comparisons using the false discovery rate (FDR) procedure by Benjamini and Hochberg (BH Method) (Benjamini et al. 1995) for the number of the population on which the test was applied. Significant units identified by ANCOVA were considered to have a force representation modulated by reward expectation, and units that also passed the BH method are stated explicitly.

#### Identifying observation modulated neurons

We started by identifying and tracking single-unit activity across multiple blocks of recorded data. We compared single-unit activity between reward-cued manual and observational blocks and again between reward-uncued manual and observational blocks for each NHP and cortices (CS1, rM1, PMd, and PMv). We tracked single-unit activity from the two manual and two observation blocks performed in a single day for each NHP. If a unit on a channel retained the same waveform overall 4 recorded blocks, we considered it the same unit. In addition, we verified this single unit activity by checking the correlation coefficient between the waveform shapes across all blocks, where a high correlation (> 0.98) indicated the same unit. We also confirmed the consistency of single-unit activity across blocks by checking the first two principal components using principal component analysis (PCA). Performing these checks allowed us to track single-unit activity with significant correlation to reward, force, or both during manual and observation tasks during cued and uncued blocks towards identifying putative mirror neural activity responding to force and reward modulation.

## Results

**Figure 3:**
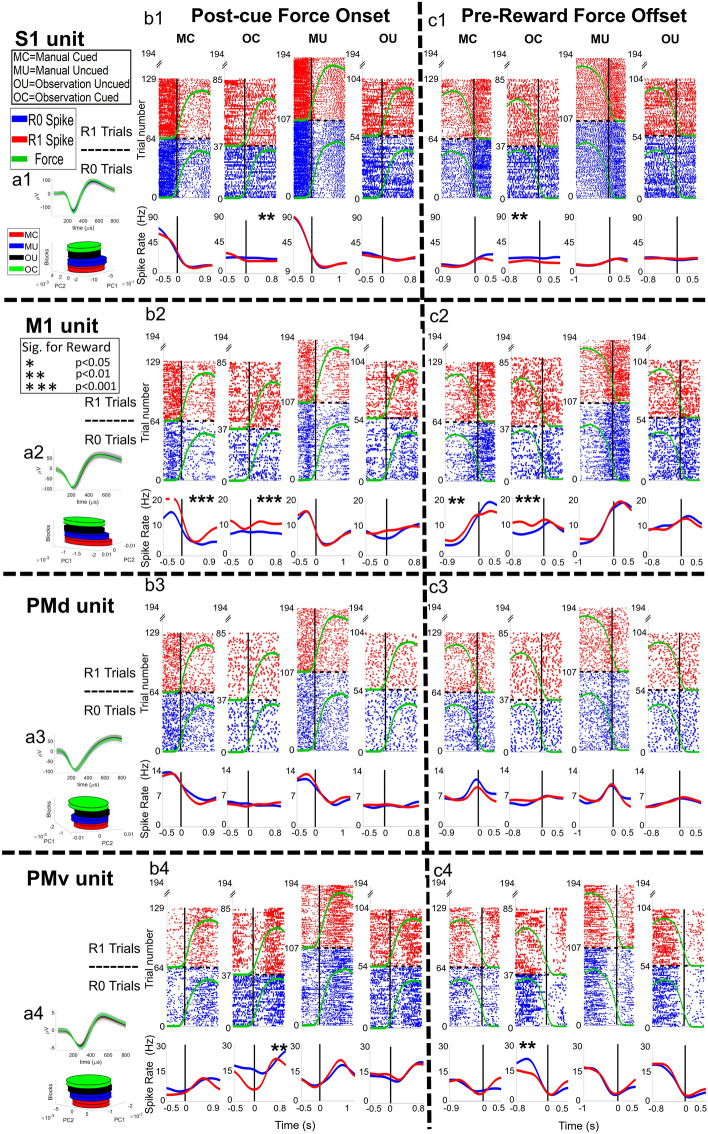
Raster plots of example units from NHP S during the manual and observational blocks. The plot numbers, 1–4, indicate the brain regions (1–4 for cS1, rM1, PMd, and PMv, respectively). The letters, a-c, before the numbers 1–4, are associated with (a) single unit waveform for the example unit, (b) force onset plots, and (c) for force offset plots. For (b) and (c), there are four plots for the manual cued task (MC), observational cued task (OC), manual uncued task (MU), and observational uncued task (OU) sequentially. The post-force onset and pre-force offset time are set as 60% of the mean force length of all trials for that task. On each subplot (b, c), the x-axis represents time in seconds. For raster plots, the y-axis represents the trial number. For spike rate plots (bottom of subplots) y-axis is the spike rate in ‘Hz’. The dashed horizontal black line on each plot divides the R1 trials from R0 trials. Below the raster plots, solid red and blue lines indicate the mean spike rate (Hz) for R1 and R0, respectively. An asterisk (*) indicates post force onset or pre-force offset spike activity is significantly (t-test, p < 0.05, p < 0.01 and p < 0.001 denoted by *, ** and *** respectively) different between R0 and R1 trials. All figures shown are significant for force modulation; see methods. See Fig. S21 for a zoomed-in y-axis version for observational tasks.

**Figure 4:**
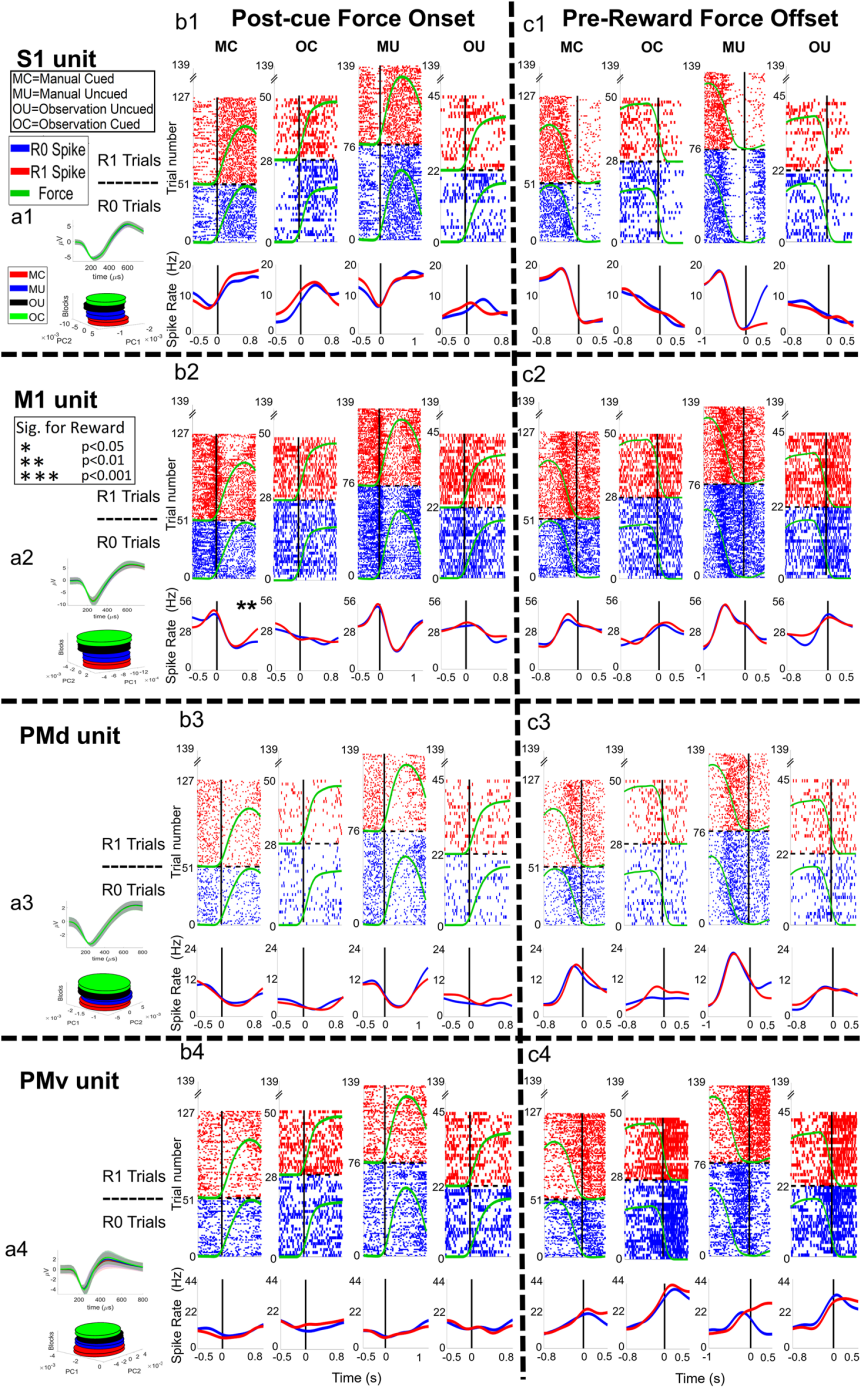
Raster plots of example units from NHP P during the manual and observational blocks. The plot numbers, 1–4, indicate the brain regions (1–4 for cS1, rM1, PMd, and PMv, respectively). The letters, **a**–**c**, before the numbers 1–4, are associated with the (a) single unit waveform for an example unit, (**b**) force onset plots, and (**c**) force offset plots. For (**b**) and (**c**), there are four plots for the manual cued task (MC), observational cued task (OC), manual uncued task (MU), and observational uncued task (OU) sequentially. The post-force onset and pre-force offset times are set as 60% of the mean force length of all trials for that task. On each subplot (**b**, **c**), the x-axis represents time in seconds; for raster plots, the y-axis represents the trial number; for spike rate plots (bottom of subplots), the y-axis is spike rate in ‘Hz’. The dashed horizontal black line on each plot divides the R1 trials from R0 trials. Below the raster plots, solid red and blue lines indicate the mean spike rate (Hz) for R1 and R0, respectively. An asterisk (*) indicates post force onset or pre-force offset spike activity is significantly (t-test, p < 0.05, p < 0.01 denoted by * and ** respectively) different between R0 and R1 trials. All figures shown are significant for force modulation; see methods. All figures shown are significant for force modulation; see methods. See Fig. S22 for a zoomed-in y-axis version for observational tasks.

**Figure 5:**
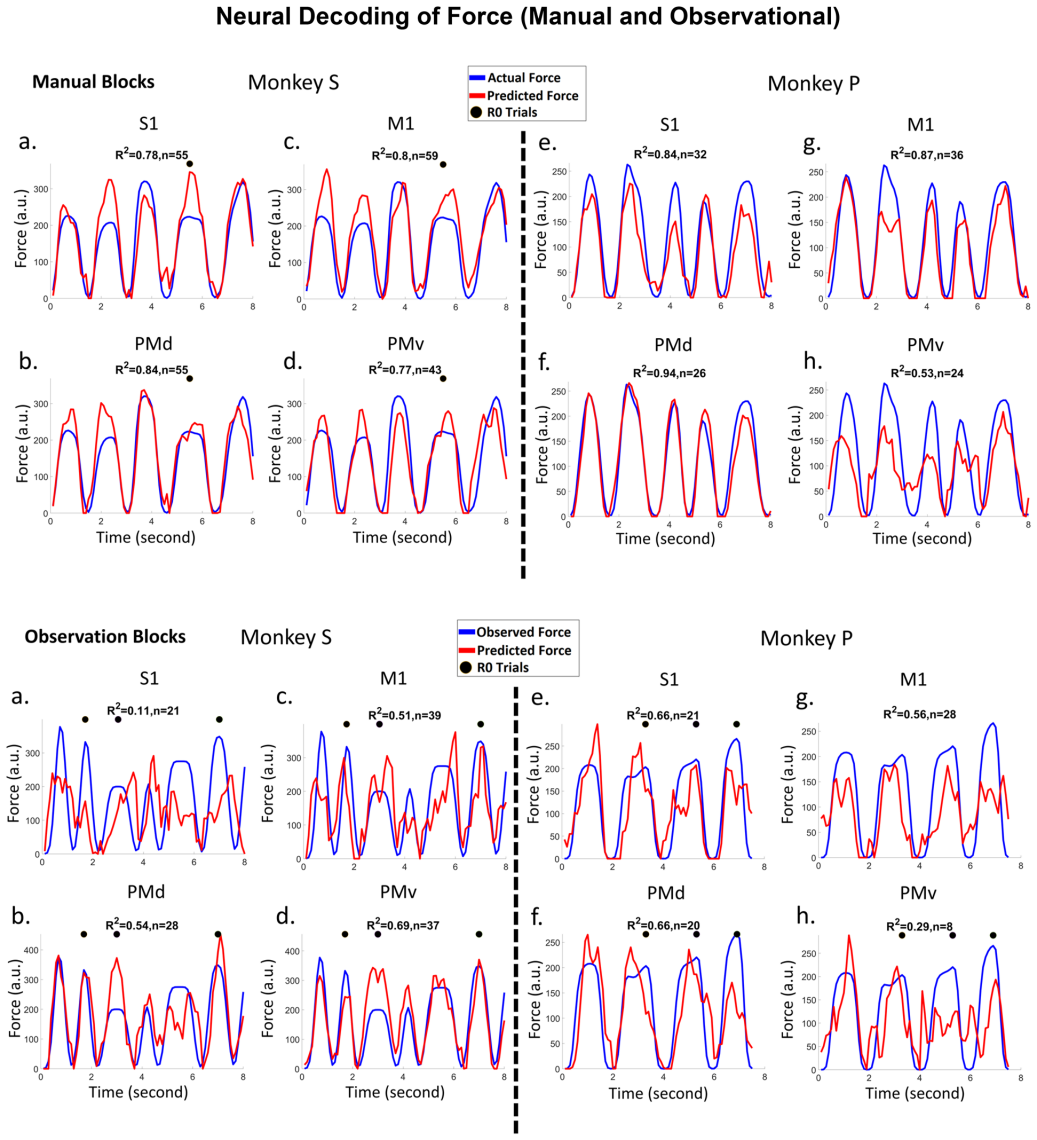
Force decoding (Red lines) from subpopulations of cS1, rM1, PMd, and PMv cortical recordings taken from manually performed blocks top two rows, and observational blocks bottom two rows. Linear regression (eq. 2) was used to predict the force from a subpopulation (see methods). The black dots on each plot indicate that the force profile was taken from an R0 trial, while all others were R1 trials. Plots *a, b, c, *and d show results for NHP S, for cS1, rM1, PMd, and PMv cortices, respectively. Plots e*, f, g, *and h show similar results for NHP P. The top of each figure shows R-squared values (R^2^) using the 10% test set and the number (n) of significant units used for regression to predict force. See Table S2 for full stats on the regression models.

**Figure 6:**
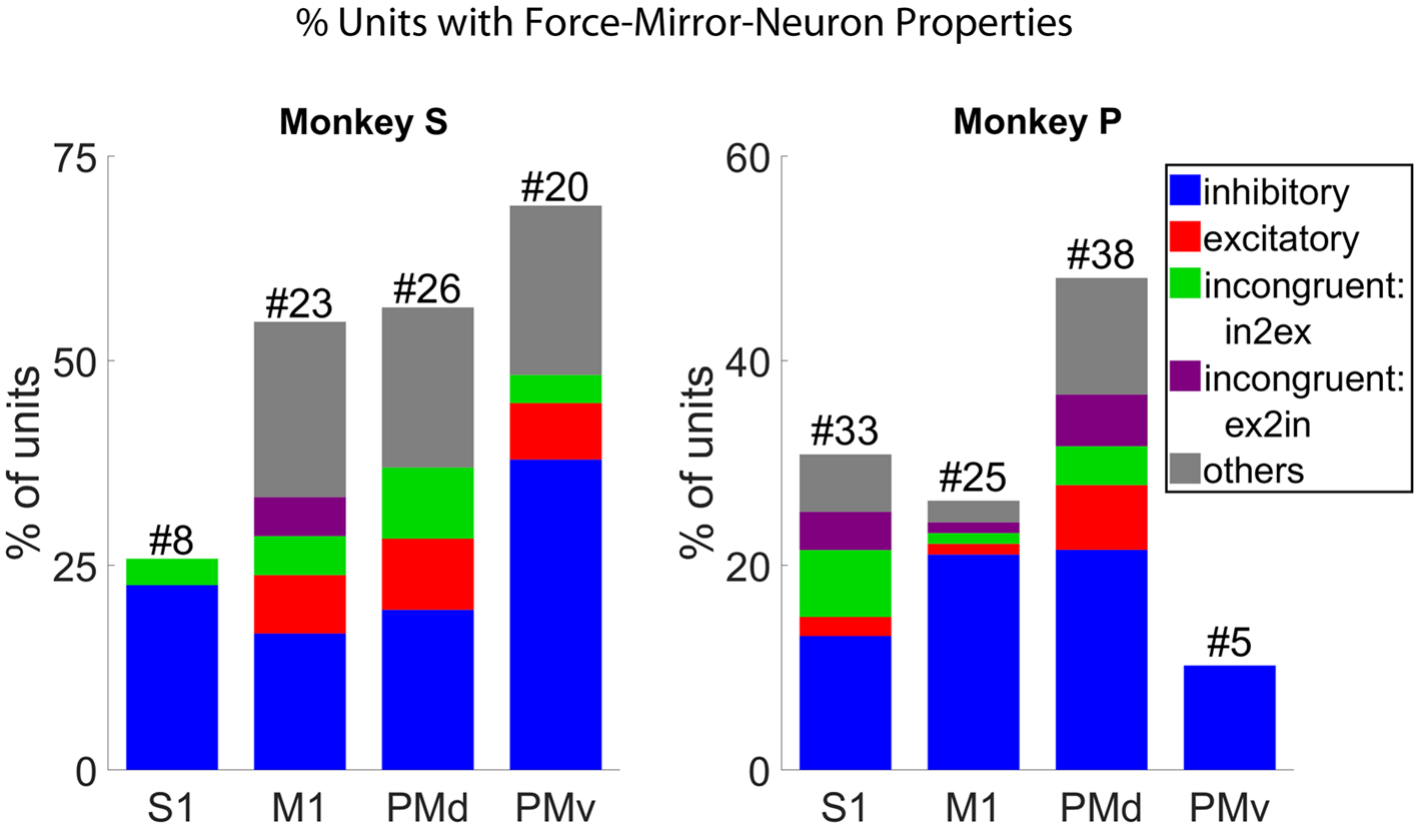
Percentage of well isolated MN single units from cS1, rM1, PMd, and PMv that showed significant (F-test, BH post-hoc adjusted for the number of units in the population) fit to grip-force during all four blocks of data (two manual and two observational) for each NHP. The different color bars represent congruent inhibitory (blue), excitatory (red), incongruent inhibitory to excitatory (green), incongruent excitatory to inhibitory (purple), and other (gray) unit responses. The percentage (y-axis) shows how many units (# at top) are significant for force from units present in all four data blocks. See Fig. S11–S12 for a breakdown of units by trial type.

The results section is structured as follows: (1) We start by presenting raw data from manual and observational versions of our isometric grip-force task showing peri-event-time-histograms (PETHs) and rasters of sample units, indicating the modulation of single units by both cued grip-force and cued reward level during manual and observational trials in the caudal S1 areas 1–2 (cS1), rostral M1 (rM1), PMd and PMv, for NHP S and P, Figs. 3 and 4 respectively. (2) We present population results from linear regression focusing on cued grip-force, showing that subpopulations of these brain regions encode grip-force trajectories (Fig. 5) even during observational trials. (3) In Fig. 6, we tested for temporal shifts between the neural representation of force, between manual and observational versions of the tasks, to determine if the same units are acting in response to the visual input, classical MNs, or predicting such input, as expected by mental simulation of the movement and associated sensory gating. 4) Subsequently, we give information on units modulated by both cued grip-force and cued reward level during the force output period of the manual and observational tasks for the same single units that are putative MNs for force modulated by reward. To be clear, the term force, when used for the observational trials, indicates the force that would be expected from the NHP if the NHP were performing the task manually, based on the visual force target cues still being presented during the observational task.

In Figs. 3 and 4b,c, we present raster plots for example units with all trials aligned to the onset or offset of the grip-force. The mean force profile (indicated with green lines on the raster plots) shows force onset and offset, plotted with arbitrary units for R0 (blue raster) and R1 (red raster) trials. The mean duration that NHP S applied grip-force was 1.4 ± 0.4 s (cued), 1.42 ± 0.42 s (uncued) during manual blocks. During observational blocks, the “force” times were 1.12 ± 0.26 s (cued), 1.16 ± 0.27 s (uncued). For NHP P, the mean force duration was 1.2 ± 0.28 s (cued), 1.45 ± 0.29 s (uncued) during manual blocks, and 1.12 ± 0.18 s (cued), 1.21 ± 0.23 s (uncued) during observational blocks, see Figs. S6–S8 for more on force level, duration and reaction times. NHP S achieved success rates of 77% (cued manual) and 82% (uncued manual) during the two manual-task blocks, while NHP P achieved rates of 58% (cued manual) and 72% (uncued manual). The number of R0 and R1 trials recorded on each block type is given in supplementary Fig. S9. The R0 and R1 mean force profiles are plotted with the same scale for comparison. All units shown in both Figs. 3 (4 units) and 4 (4 units) were significant (F-test, BH corrected for p = 0.05 for the number of units within the given brain region) for grip-force. For each NHP and cortices, the number of units recorded is given in supplementary (Fig. S2), min ~ 50 units and max ~ 170 units. In Fig. 3 ax (x = 1, 2, 3 and, 4) we show the mean spike waveform with standard error (shaded) for a single unit (top) and its PCA space (bottom) for all 4 blocks, manual cued (MC), manual uncued (MU), observational uncued (OU) and observational cued (OC), which were all recorded on the same day and in the above order for each NHP. Each raster subplot (b–e) shows activity for force onset (left) and force offset (right) periods.

Units in Figs. 3 and 4 were chosen to represent the variety of responses we saw in the population. We utilized 100 ms bins during the post-force onset spike activity from the point when force sensor values crossed above 50 (a.u.) and pre-force offset activity until the reading went below 50 (a.u.). Fifty was the same value used for the task logic and was chosen based on experience with these NHPs performing this task to minimize false starts without missing movements. We collected p-values from t-tests on Spearman Rank correlation between the binned (100 ms) spike rate and corresponding reward levels (1 for R0 and 2 for R1), indicating significance with an asterisk (*). For example, in Fig. 3b1,c1, we see a cS1 unit that decreases its activity at or just after the onset of “force” and is suppressed around the force offset period. Our hypothesis is that this unit, and other suppression like it, is due partly to these units being connected to extensor muscle groups of the forearm and thus showing inhibitory spike activity during gripping action (see Figs. S17 and S18 for support of this). Other hypotheses would be that the suppression is related to an efference copy or transmission of MN activity in M1 to S1. In addition, the unit in Fig. 3b1,c1 shows modulation by reward. Similarly, in Fig. 3b2,c2, we see an rM1 unit with a similar response to the cS1 unit, where the rM1 unit is suppressed at force onset and activated at force offset with some reward modulation as well. In Fig. 3b3,c3, a PMd unit is shown that is suppressed before force onset and has its peak activation pre-offset. Monkey P data was not as responsive to reward activity utilizing the spearman rank test as Monkey S. The Fig. 3 responses shown for cS1, rM1, and PMd resemble extensor patterns of activation. In contrast, the PMv response shown resembles a more flexor typical response^42,43^. In Fig. 4 we have chosen units that show some other responses seen in the population for both NHPs. The observational response to “force” was weaker than the responses to actual force during manual trials and did not always align with the force onset and offset times precisely. For additional raster plots on example units during grip force observation, please see supplementary Fig. S10. All units seen in Figs. 3 and 4 had significant fits to grip-force (F-test, p < 0.05). This F-test was performed on the regression model (Eq. 1) to test whether it was a better fit than a degenerate model, which consisted of only a constant term. Figures 3 and 4 show a total of 8 example units; 1 from each cortical region (rM1, cS1, PMd, and PMv) for each NHP, P, and S, from reward cued and uncued blocks.

### Grip-force decoding during manual and observational trials

As described in the Methods section and further in the supplementary section (Fig. S5), data blocks were analyzed using a two-step process. We identified significant units related to force using the simple linear model Eq. 1, ${y}_{f}=a{\mathrm{X}}_{fr}^{{n}_{i}}+b$, where ${y}_{f}$ is the vector of grip-force trajectory, $a$ is the vector of regression parameters that multiply unit ${n}_{i}^{\prime}s$ firing rate, where we used the associated F-statistic with a (p < 0.05) for significance determination. Note, we utilized neural data around a given grip-force timepoint, from − 0.5 to 0.5 s in 100 ms bins for this regression, thus 10 bins. We extracted single units with activity that improved force decoding. Model 1 variables ${\varvec{a}}$(10 per unit) and $b$ were fit using least-squares estimates from 90% of the recorded data (see Fig. S5 for data split cartoon). The remaining 10% of the data was used as a test set to verify the performance of force decoding. The R-squared values for decoding on the test set of forces are shown below. We found high prediction accuracies for both NHPs. The results shown in Fig. 5 are force decoding prediction from cS1, rM1, PMd, and PMv cortices for cued manual blocks, top two rows, with the corresponding observational blocks seen in the bottom two rows. Note that intertrial intervals have been clipped out for presentation purposes.

The manual tasks generally had higher levels of prediction as compared to the observational versions. We detected the units consistently present in all four data blocks (two manual and two observational) and those significant (F-test, BH adjusted for p = 0.05 and number of units in the population) for grip-force fit in all data blocks. For NHP S, we found 31 units in cS1, 42 in rM1, 46 in PMd, and 29 in PMv that were consistently present in all four data blocks. For NHP P, the numbers were 107 in cS1, 95 in rM1, 79 in PMd, and 49 in PMv. Below in Fig. 6, we show the total percentage of these units from each region that showed significant linear regression model fit under both the manual and observational versions of the task that are the putative MNs. We used the peak value from the absolute correlation coefficients between grip force and each of the 10 bins of spike rate (100 ms from − 0.5 ms pre grip force spike rate to 0.5 s post, described in the “Grip-force trajectory prediction” section of the methods), to detect inhibitory or excitatory activity. We considered a unit’s representation excitatory if the actual value of that peak absolute correlation was positive otherwise it was considered inhibitory. We detected congruent units among the significant units that showed similar activity in all four data blocks of either inhibitory (blue) or excitatory (red) spike rate in relation to grip-force. We hypothesize that the units showing inhibitory activity during grip-force application are connected to the extensor muscle groups of the forearm, which relaxes during gripping activity and show evidence for this in Fig. S17–S18. Incongruent units showed opposing behavior between manual and observational tasks, such as excitatory during manual, and inhibitory during observational (purple), or inhibitory during manual and excitatory during observational (green) in Fig. 6. Other (gray) units did not follow any simple pattern during all four data blocks, as seen in Figs. S11–S12. The number of units for each possible combination of inhibitory or excitatory spike activity during grip force for four data blocks are given in the supplementary (Fig. S11 for NHP S and Fig. S12 for NHP P) section. Monkey S showed what one might expect PMv > PMd > rM1 > cS1, whereas NHP P’s data was not in this clear expected order and could be due to the placement of the electrode arrays due to the vasculature-imposed restrictions as seen in Fig. 2. However, in both NHPs, the largest single group of MNs appear to be congruent and inhibitory for our task (blue bar sections).

### Distribution of reactive vs. predictive mirror neurons

**Figure 7:**
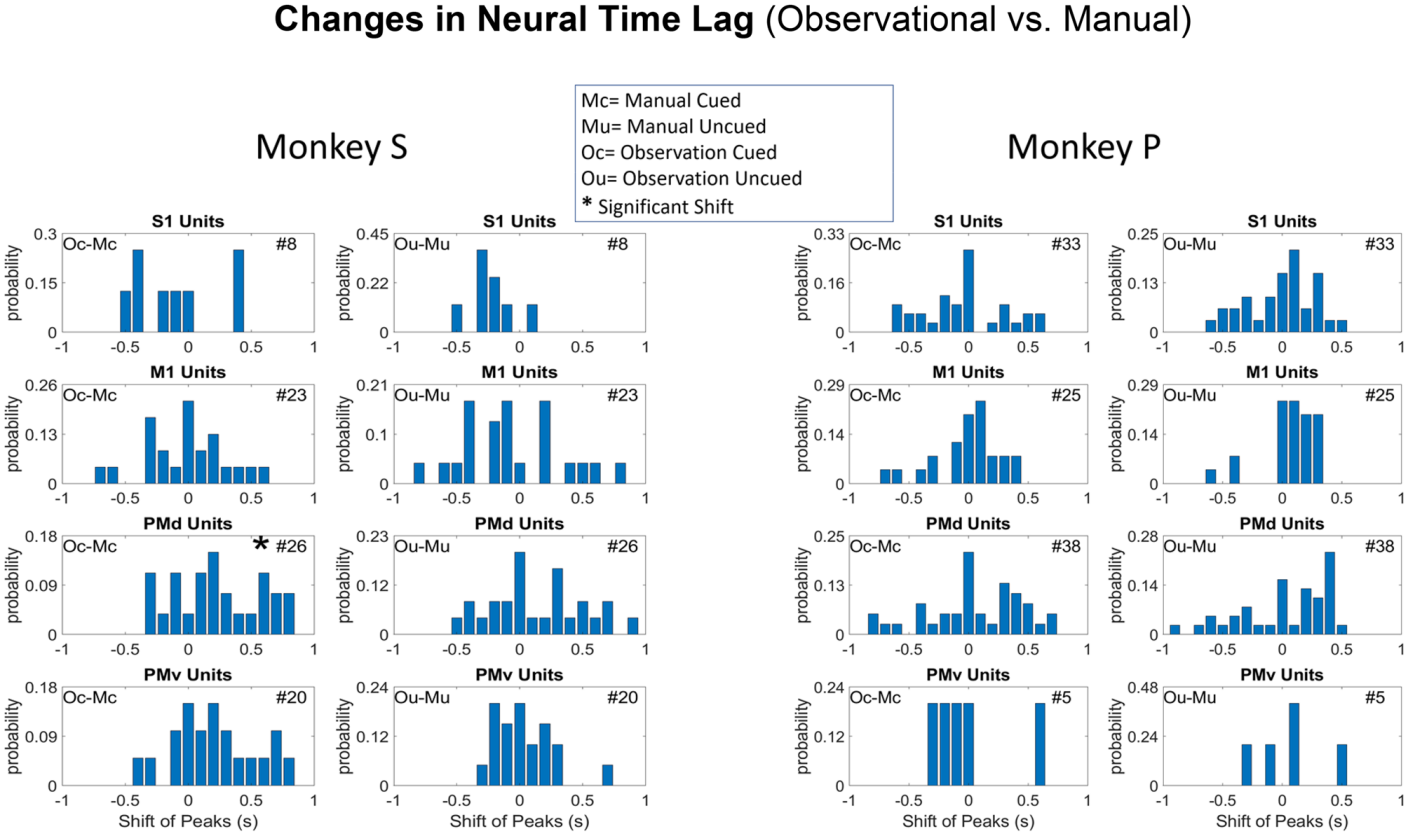
Changes in neural time lags where neural data best correlate with peak grip-force. Bar plots showing the shift of peak correlation between similar (cued/Uncued) Manual and Observational blocks for cS1, rM1, PMd, and PMv cortices (cortex is labeled on the title of each subplot). The shift from a block type to another block type (manual-to-observational) is at the top right of each subplot for each NHP (Mc = Manual Cued, Mu = Manual Uncued, Oc = Observation Cued, Ou = Observation Uncued). The number of units used is indicated in the upper right corner of each subplot. The left column for each NHP shows the shift between cued blocks, and the right column shows the shift between uncued blocks. An Asterisks (*) symbol before the number of units represents a significant (p < 0.01) shift in the histogram from manual to observational tasks. The probability of unit shifts was calculated by subtracting the peak correlation time bin position for manual from the observational tasks for the same unit. For more on this correlational analysis see Figs. S13 and S14.

This section's analysis was conducted to determine if there was a significant shift in the time lag between the neural correlates of force between manual trials and observational trials. One would expect the neural data responsible for force production or imagining force production to lead the force output, whereas classical MN activity would lag the viewed “force” output. However, this may not be the case for predictable movements, such as those used in our task, as described by others^44^, where MNs can still lead the observed task. In Fig. 7, significant force units (F-test, p < 0.05, Eq. 1) in all block types were plotted in the time bin where their correlation coefficient with force was maximum in absolute value, either negative or positive. We asked if these distributions significantly (signed Rank test, p < 0.01) deviated from zero between the manual and the observational versions of both the cued and uncued tasks. There were no significant shifts in the histograms for NHP P. NHP S showed one task with significant shifts for PMd during the cued tasks. For each plot, the zero-time shift shows the probability of a unit not shifting in time between manual and observational. The positive differences are the classical MN response where observational neural activity lags the visual information. In contrast, the negative time shifts represent the probability of the observational neural responses being earlier or more “predictive” of the moment than during the manual trials. For more on correlational analysis, see Figs. S13 and S14.

### Force and reward modulation of single mirror neurons

**Figure 8:**
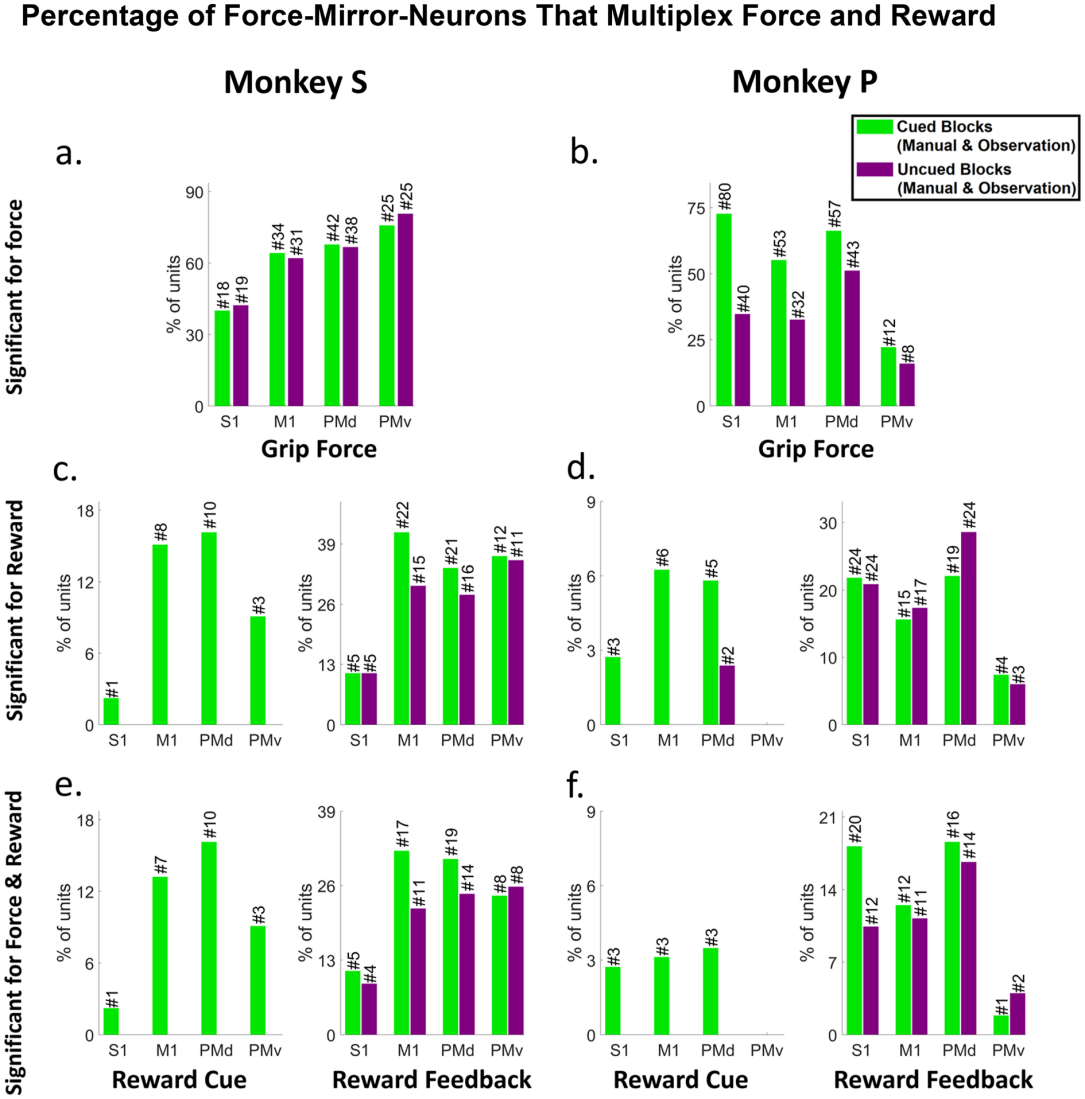
Mirror Neurons in cS1, rM1, PMd, and PMv Multiplex Reward and Grip-force. The percentage of units with significant modulation via either grip-force (F test, BH method for p = 0.05), reward (post-cue or post-reward, t-test, BH method for p = 0.05), or both, during manual and observational blocks of a similar type (e.g., cued or uncued). The number of hypothesis tests for the post hoc BH method was the number of MNs in that brain region under study, seen above each bar. Green represents units for cued blocks and Purple for uncued blocks. Each subplot shows the percentage of significant units between cued/uncued units showing such activity during both the manual and observational blocks for Force (plot a and b), Reward (plot c and d), and significant for both force and reward (plot e and f) during separate times in the task. For subplots c, d, e, and f, the left plot shows units for post reward cue, and the right plot shows units for post reward activity after reward delivery (150 ms) was completed.

**Figure 9:**
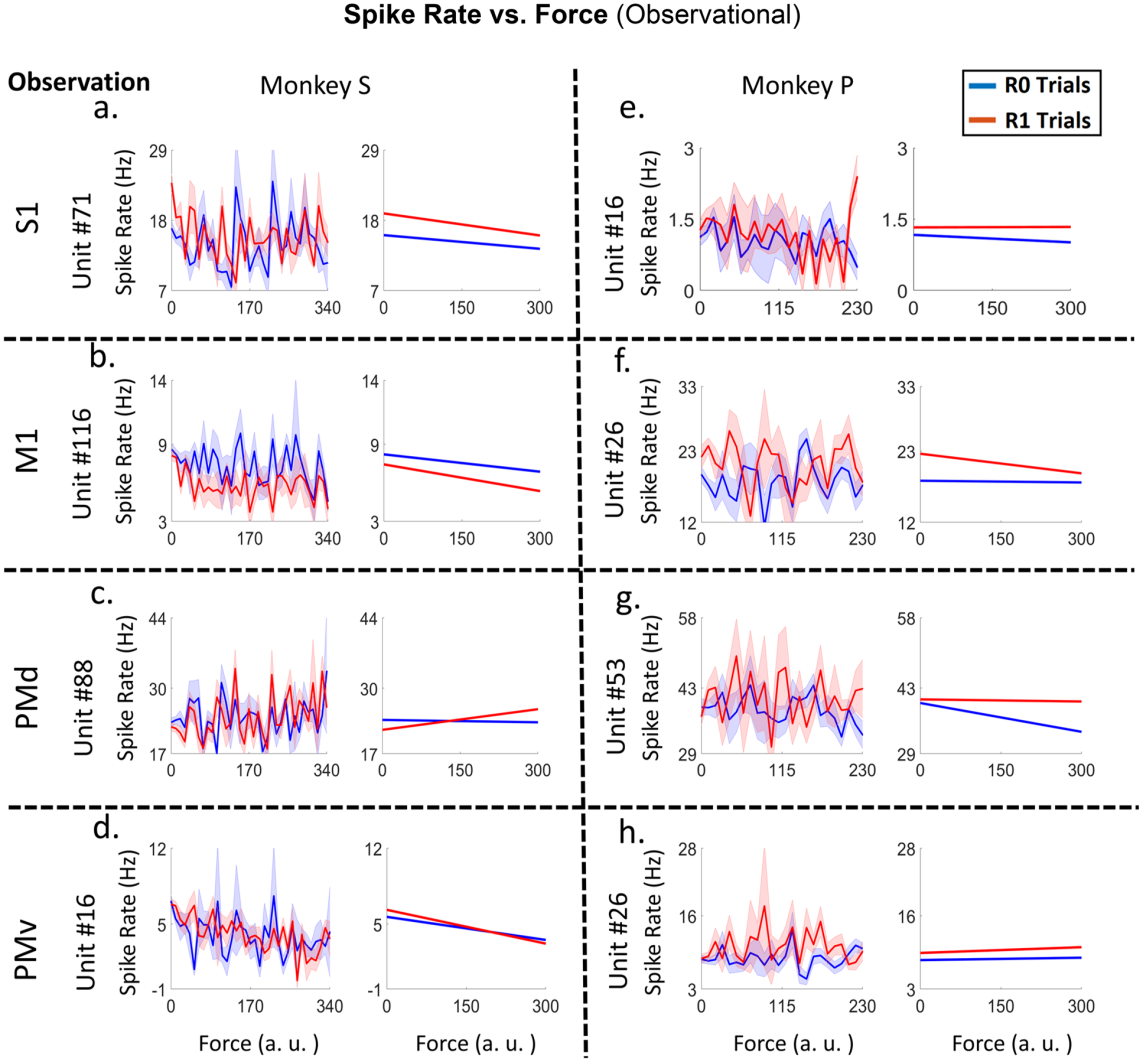
Plots of spike rate vs. force (left subplots) and their linear tuning curves (right subplots), for example, units from cS1 (plot *a, e*), rM1 (plot *b, f*), PMd (plot *c, g*) and PMv (plot *d, h*) cortices of both NHPs (for NHP S plots *a, b, c,* and *d* and for NHP P plots *e, f, g,* and *h*). The units presented had significant differences between R0 and R1 groups (ANCOVA, F-test, p < 0.05) force tuning curves during observational blocks. Red lines indicate rewarding trials (R1), and blue indicates non-rewarding trials (R0). See Fig. 10 for population results and Fig. S15 for the manual version of this figure.

**Figure 10:**
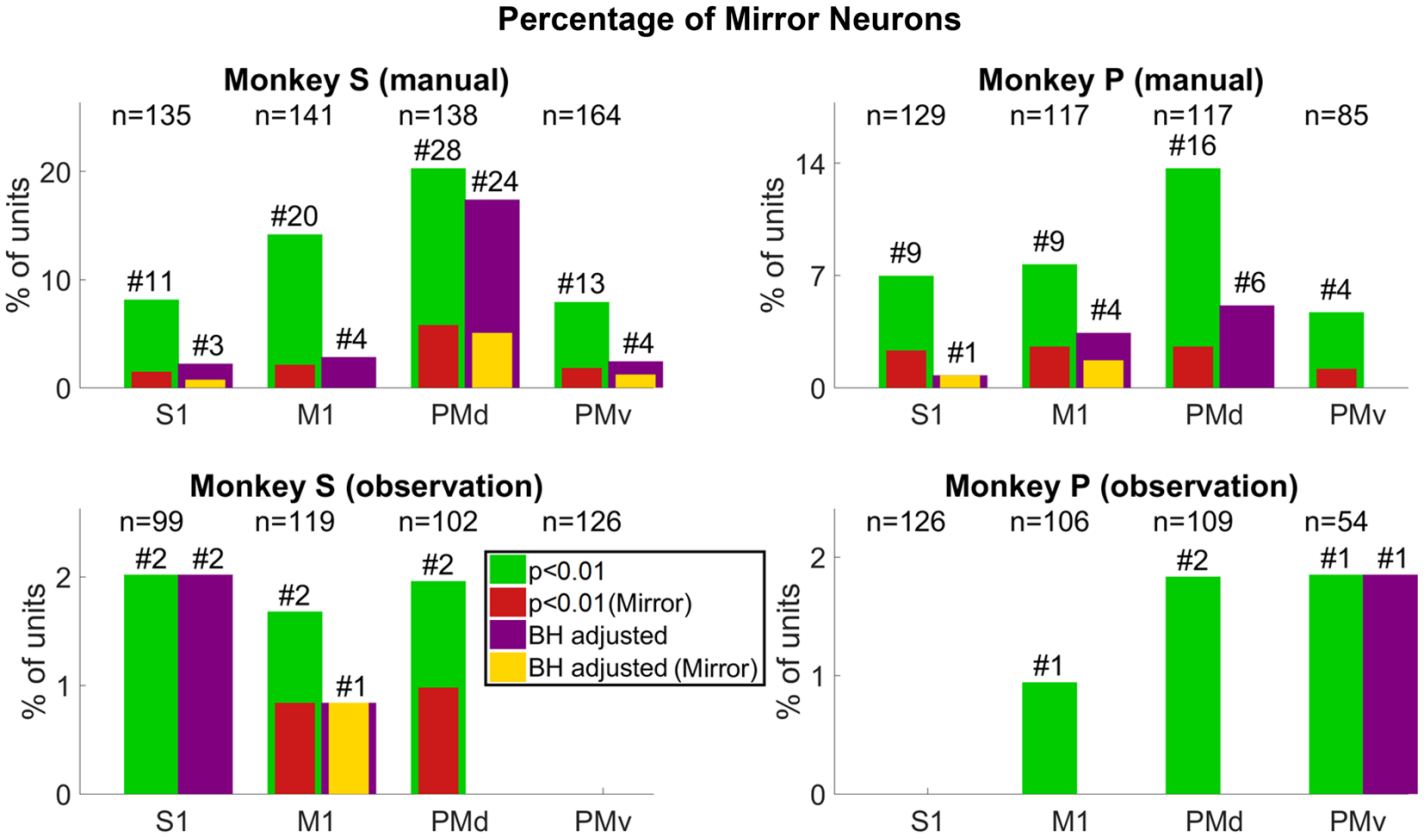
The percentage of single units with force tuning curves that are significantly modulated by reward level. The titles represent which NHP and block type are plotted. P-values were adjusted using the Benjamini–Hochberg method (BH method), correcting for false discovery rate, where the correction was applied using p = 0.01, and the number of hypotheses tests applied was the number of units in the population under study. The y-axis is the percentage of units, and the x-axis represents the cortical region. The green bar is showing the number of significant units (p < 0.01, ANCOVA), and red shows the MN population among them. The purple bar is showing the significant units after the BH method was applied. The yellow bar inside the purple is showing the percentage of putative MNs after the BH method. The number after the ‘#’ sign on top of each bar shows the number of significant units for that given case, and the number presented with ‘n’ is the total units recorded for that case.

Figure 8 shows that a good percentage of units show MN activity encoding force during manual and observational trials (Fig. 8a,b). The percentage of units showing this MN activity is generally greater when a reward value cue is shown before movement as compared to the uncued version of the task. We also show the percentage and number of units that were significantly correlated with force or reward separately Fig. 8c,d, and units that were significant for both Fig. 8e–f, but not simultaneously, which would be comodulation and is shown in Figs. 9 and 10. Notice that generally, only the cued task shows significance during the reward-cue period compared to the uncued task, which demonstrates the reward-cue modulation of the neural activity. Units in Fig. 8 were significant under both manual and observational trials. Single unit activities were tracked across manual and observational cued and uncued blocks to determine the significance of their correlation with force under these different conditions. That is, a unit that was present during both cued manual and cued observational blocks was checked for significant correlation with force and/or reward and included in Fig. 8 only if it was significant for both manual and observational trials towards the possible discovery of MNs. The same procedure was performed for uncued manual and observational blocks separately. The plots in Fig. 8 show the % of units (left y-axis) and the total number of units (top of each bar) for each category identified from cS1, rM1, PMd, and PMv recordings. The green bars are for reward level cued trials, while the purple bars are for reward level uncued trials. The analysis windows used for reward were, for post-cue from 0 to 0.5 s, and for post-result from 0 to 0.5 s, while for force significance, the window was 0.5 s starting at pre-force-onset to 0.5 s after post-force-offset. Note that these time windows were not overlapping; thus, Fig. 8 is not describing the results of force tuning modulation by reward, which is described in Figs. 9 and 10.

### Comodulation of force tuning curves by cued reward level

In Figs. 9 and 10 we ask questions about the modulation of the force tuning curves of MNs by cued reward level. Force tuning curves for R1 and R0 trials, as well as significant differences between their slopes, were calculated as described in the methods using MATLAB’s ANCOVA function. F-statistics were conducted on the reward group*force interaction, which expresses the difference in slopes, and the p-values for those interactions were collected. Figure 9 shows the force tuning curves, for example, units recorded from reward-cued observational blocks for each brain region (see Fig. S15 for the manual version). The relationship between R1 and R0 trials as spike rate varied with force can be seen in Fig. 9. This is compared to the previous sections when force decoding meant the neural activity could be used to determine the force level. In contrast, here we look at the change in firing rate as a change in force level and its modulation by reward (encoding). The smoothed spike rate against force is shown in the left subplots, while the line plots to the right show force tuning curves obtained from the ANCOVA. The two example units from each NHP, S and P, had significant differences between R0 and R1, in agreement with our previous rM1 results for manual trials^8^. Again, results in Fig. 9 are for observational trials only, and manual versions can be seen in Fig. S15.

In Fig. 10 we show results indicating the degree to which cS1, rM1, PMd, and PMv not only multiplex information on both force and reward expectation during both manual and observational tasks, but how reward expectation modulates the force tuning functions that is, the degree of comodulation. Figure 10 green bar plots indicate the percentage of single units with significant grip-force tuning curves (ANVOCA, p < 0.01) for the NHP and task type indicated at the subplot title. Purple bars show the subpopulation of these units (green bars) that pass a rather stringent post-hoc false discovery rate correction (BH, p < 0.01, # of hypothesis corrected for is the number of units being considered for tuning curve testing in that brain region). Red bars indicate the subpopulation of the grip-force units that are modulated for both task types (manual and observational), that is, MNs. Likewise, yellow bars indicate the MN subpopulation after the post-hoc test (BH, p < 0.01, # of units). Figure 10 indicates that Comodulation of force by reward is most likely significant in manual tasks, but support for the MN activity during observation of this comodulation of force by reward is rather weak (1–2% max), which could in part be due to the lower number of trials recorded during observation compounded by the lower firing rate. See Tables S3–S4 for more information on the ANCOVA results.

## Discussion

The main outcome of this work is clear support showing that MN’s can encode grip force (Figs. 5 and 6 and Tables S1 and S2), a non-kinematic variable in cS1 that is areas 1 and 2 as well as in rM1, PMd, and PMv (see Fig. 2). Secondly, that the MN activity during observational trials, as compared to manual trials, can shift their temporal relationship predicting/responding to visually cued force about equally in either the predictive or responsive time lag directions seen in Fig. 7. Thirdly, we have shown that the firing rate in each of these regions is also modulated by reward level expectation during the post-reward-cue period for reward-level-cued trials and during the post-feedback period for both cued and uncued reward level trials seen in Fig. 8. Fourthly, that MNs’ activity of visually cued grip-force can coexist (multiplex) within single units that also code for reward level seen in Fig. 8. Finally, we have shown evidence, albeit weak (~ 3–4% of MNs for manual and ~ 1–2% of MNs for observational tasks), that the neural grip-force tuning functions can be modulated by reward expectation (comodulation) in the brain regions under study (cS1, rM1, PMd and PMv) during both manual and observational trials Figs. 9 and 10.

To the best of our knowledge, we are reporting the first evidence of MN responses to expected or visually cued effort (grip-force) within these sensorimotor regions at the single-unit level. As the NHPs had a great deal of experience with the cued grip-force task, we expect the mirror responses were due to the visual cueing of the force targets that the NHPs had come to understand based on their manual training. In Fig. 7 we showed that the shift in peak correlation between cued force and neural time lag could be in either the direction expected for motor production, or its suppresion^4^, or that expected from an efferent copy, or even some form of action understanding which would be more in line with the initial MN work^1^, and the former more in line with movement rehearsal^2^. For issues with the action understanding perspective, see^45^. We predict that such force MN activity could be seen in more natural settings if the context was understood by the NHPs, such as lifting heavy vs. light objects they were familiar with, or when the NHP squeezes a deformable object, or observes such a grasp, again with the knowledge of the object’s physical properties. However, further work is needed to show that such activity exists under more natural conditions within each of these regions. It should be noted that this type of prior knowledge is necessary for MN activity patterns is not new. Mirror neurons that encode both subjective value and grasp-force require the NHP’s familiarity with the object to have formed a subjective value^46^. This could occur through reinforcement learning, where a broadcast reward signal could gate synaptic plasticity in conjunction with spike-time-dependent-plasticity rules^47–51^.

There are several possible causes for the MN activity we have presented in S1. We focus on S1 as there is less evidence for single unit MN activity in this region compared to the others (PMv, PMd, and M1). This cS1 MN activity could be due to sensorimotor simulation, including an efferent copy of expected sensory information. The cS1 MN activity could be due to a built-up association from extensive practice the NHPs had with the manual version of the tasks, via Hebbian Learning^47^, which can be carried out by spike-time-dependent-plasticity and should involve PKMζ^48,52,53^ based long term potentiation. cS1 MN activity could be part of the sensorimotor plan that is being suppressed, as has been found in M1^4^. Tracer injections into the grasp region of M1 led to staining in areas of S1, including 3b, 1, and 2^54^. This indicates at least one path that MN information could flow from M1 to S1.

We have shown that reward modulation can occur post-cue when the cue indicates the reward value of the current trial and post-feedback when the reward level that was cued is delivered. When there is no reward cue given in uncued blocks, there is no “post-cue” reward modulation as expected. There is still post-feedback modulation in each of the 4 brain regions, and again during both manual and observational trial types. Based on the evidence we have presented, we see that cS1, rM1, PMd, and PMv contain units that encode reward expectation and reward itself, which has been shown for the current tasks, and for others in rM1^6–8,23^, indicating this reward signal may be a generalized broadcast signal to regions involved in sensorimotor planning and movement production. This reward signal appears during observation of movements, in a manner that can be predictive, as expected during mental simulation^2^, and responsive to the visual stimuli, as expected for classical MNs (Rizzolatti et al. 1996; Gallese et al. 1996). In addition, we have now shown such reward modulation simultaneously for rM1, cS1, PMd, and PMv during both manual and observational trial types for the same single units tracked between tasks.

There are several limitations to the work presented here that should be addressed in future work. EMGs were not available for these particular datasets that allowed us to track single units between manual and observational trials, and therefore we cannot state with certainty that the NHPs were not activating their arm muscles covertly. However, the NHPs were not making obvious movements and did not have access to the force transducing handle. Furthermore, our previous studies found no correlation between EMGs and cursor motion in observational trials of a reaching task^7^. In addition, we show in Fig. S16 that for both NHPs, there was no significant EMG activity during observational trials on different days when the EMG signals were usable. The grip-force output in the current task was generally phasic with a bell-shaped profile, even if the cued amplitudes were different, the force profiles were still stereotyped, and so it is possible that some other phasic neural response was allowing the regression models to predict force output, which was bell-shaped, by some non-force related phasic response, such as that related to the cued reward information. However, the regression models did not fall apart in the uncued reward level task, which indicates that at least cued reward does not explain our force decoding results. In addition, when we analyzed the time series of just the peak force amplitude of each trial, we still obtained positive results showing MNs for force, although not as strong as when using the fuller dataset including the force trajectories (see Fig. S19). Additionally, in Fig. S20 we show the significance for several measures on the manual data between R0 and R1 trials post data pruning to keep the trials between these two categories statistically indistinguishable.

Previous work has suggested that MN activity is not seen in S1^55,56^; however, in Lemus et al.^55^ they did see non-frequency discriminative activation of S1 due to auditory stimuli. Others have suggested that S1 is, in fact, modulated in a MN manner utilizing fMRI^57^. Histological studies where know MN brain regions were injected with tracers showed minimal evidence of S1 staining^58^. In Fig. 7 of Gharbawie et al.^54^ there is clear evidence that injections into the grasp region of M1 lead to diffuse weak staining of area 3b, with increased staining in areas 1 and 2. The data we present for MN encoding grip force is apparent and significant. The results show that these MNs multiplex cued reward level, or reward expectation and force; however, the results for comodulation of force tuning curves by reward were less convincing but still significant. Even though S1 activity could predict grip-force output, should that be taken as proof of mirror activity? The cS1 activity may be some form of sensory gaiting, which is known to occur during passive arm movements in NHPs^59,60^ and observation of movement in NHPs and humans^61^. In Sharma et al.^56^ utilizing fMRI on NHPs, there was no clear activation of S1 tactile areas to observed touch. In their work, the NHPs were sedated with ketamine to define the tactile regions, likely decreasing the brain regions that could be stimulated in an awake subject. Additionally, as the NHPs were sedated and “blindfolded”, there would not have been any specific visual-tactile association, which may be needed to form certain “mirror” responses. During the awake portions of the research, the NHPs had to fixate on a point while video clips were shown. However, the NHPs did not necessarily have to pay attention to the video, and no understanding was tested that they did. In short, there is still debate as to the existence of mirror activation of S1.

The work presented here has a practical application past fundamental neuroscience. We believe it is essential to continue gaining a better understanding of encoded information, such as reward, within the sensorimotor cortices toward the development of a closed-loop brain-machine interface (BMI) for the restoration of motor control and beyond, such as towards a better understanding and tracking of the psychological state of the individual^62^. BMI neural signals are often recorded and decoded from a subset of cS1, rM1, PMd, and PMv. At the same time, sensory feedback is obtained either by natural vision or stimuli routed to cS1, such as via the thalamus^63^ for somatosensory feedback. It has recently been shown that reward expectation can change directional and force-based tuning curves in rM1 and cS1^8,23^, and here we have shown that all of these brain regions are influenced by reward expectation, effort, or sensory feedback on expected effort. Therefore research into these relationships is warranted. Differentiating between mirror activity in these regions and intentional activity for movement is vital towards making BMIs more stable for the users intended movements compared to observed movements, such as when working in team environments with others’ arms and hands seen in the common workspace. Our future work will address these issues.

## Supplementary Information

Supplementary Information.
