# Supplementary material for: Mirror neurons are modulated by grip force and reward expectation in the sensorimotor cortices (S1, M1, PMd, PMv)
Source: Sci Rep. 2021 Aug 5;11:15959. doi: 10.1038/s41598-021-95536-z (PMC8342437; doi:10.1038/s41598-021-95536-z)
Supplement: Supplementary file 1 — Supplementary Information. [file 41598_2021_95536_MOESM1_ESM.docx]

**SUPPLEMENTARY INFORMATION**

**Mirror Neurons are Modulated by Grip Force and Reward Expectation in the Sensorimotor Cortices (S1, M1, PMd, PMv)**

**Abbreviated title:**

Md Moin Uddin Atique^[[1]](#footnote-1)^ and Joseph Thachil Francis^1-2 *^

**Correspondence:** Joey199us@gmail.com

**Acknowledgments**: Research was supported by NIH 1R01NS092894-01, NSF IIS-

17 1527558, DARPA REPAIR Project N66001-10-C-2008.

Department of Biomedical Engineering, Cullen College of Engineering, The University of Houston, Houston, TX 77204, United States.

2 Department of Electrical and Computer Engineering, Cullen College of Engineering, The University of Houston, Houston, TX 77204, United States.

^*^ Corresponding author - jtfracis@uh.edu

**Supplementary Information**

**Autocorrelation of the Reward Level Target Sequence:**


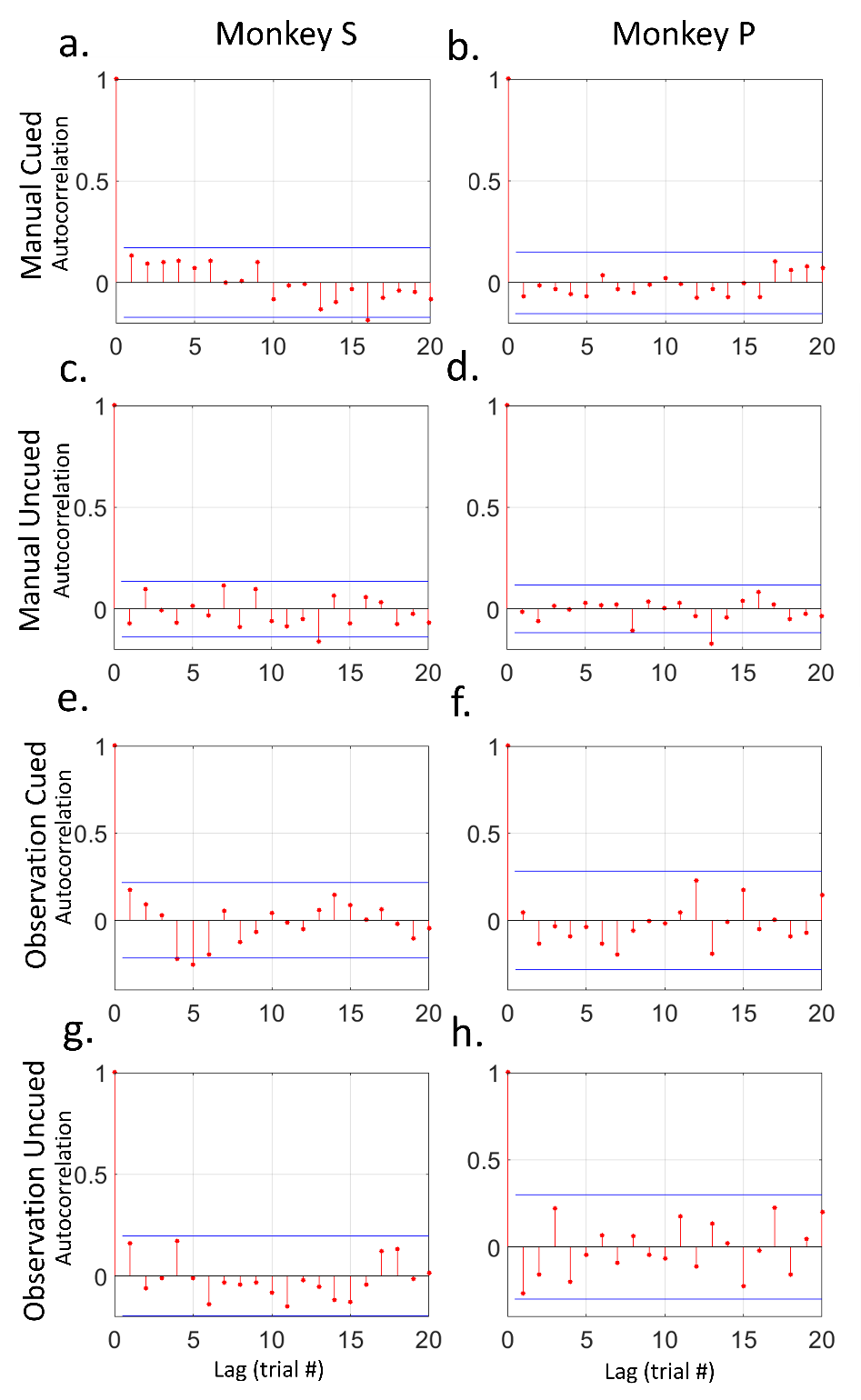


**Figure S1**: Autocorrelation of the reward level target sequence (R0 and R1) of trials for all data blocks. Plot a, c, e, and g are for NHP S, and b, d, f, and h are for NHP P. The block type is given on the right side of each row. The x-axis represents lag in trial numbers, and the y-axis represents the corresponding autocorrelation.

**The number of Single Units Recorded for Each Cortex:**

The figure below shows the total number of single units recorded in all the cortices used (S1, M1, PMd, and PMv) and each block type (MC, MU, OU, and OC). The block types are color-coded, as seen in the legend in the right subplot. The left subplot shows the number of units from NHP S, and the right shows it for NHP P. The x-axis is the cortical region, and the y-axis is the number of units.

**
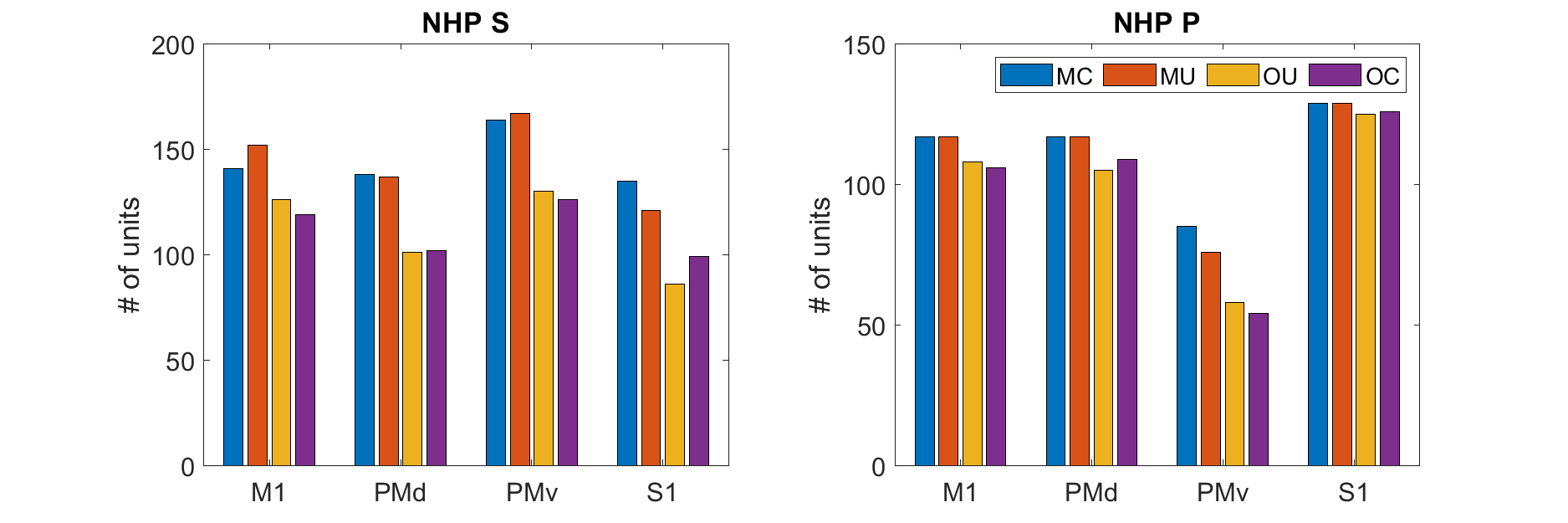
**

**Figure S2:** The total number of units recorded in all four cortices (S1, M1, PMd, and PMv) for both NHP S (left) and NHP P (right). For each cortex, there are four columns for the four data block types (MC, MU, OU, and OC) that are color-coded as in the legend in the right subplot. The y-axis represents the number of units.

**Decoded Force R-square Value with and Without Smoothening (Fig.S3):**

We applied MLR (Multiple linear regression) to decoded grip-force from smoothened spike rates (shown in the main text figure 5). To show that the smoothening was not playing a major role in our decoding results, we also tested the square root transformed spike rate without smoothing to decode the force following the same procedure described in the method section. The following figure shows the R-square values of force decoding with (red) or without (blue) smoothening. As can be seen the results are similar and even better for the non-smoothed version at times.


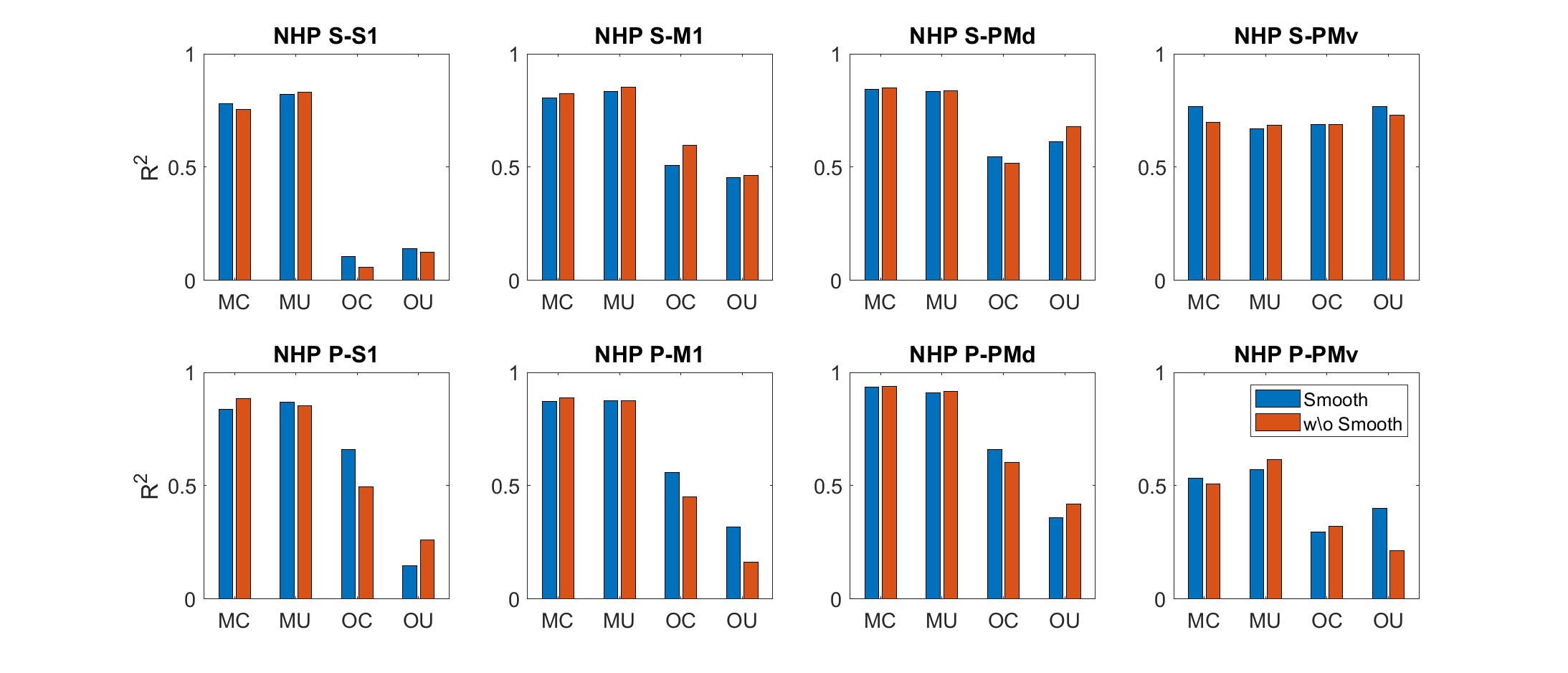


**Figure S3:** The R-square of the decoded grip-force with (blue) or without (red) smoothening was applied on the spike rate data. The rows of subplots show data from S1, M1, PMd, and PMv cortices of NHP S, and the bottom row is the same for NHP P.

**Grip-force prediction without Square root transformation (Fig.S4):**
In the following figure S4, we show a comparison between the grip decoding accuracy with R-square values between decoded grip-force on the test set of data and the actual grip-force applied (manual) or observed (observational). The bar plots showed that the R-squared does not deviate much when a square root transform is applied (blue) on the spike rate from the case when the transformation was not applied (Red). Figure 8 in the main text shows that NHP S had a higher percentage of significant units, especially in M1, PMd, and PMv cortex, due to reward cues compared to NHP P.


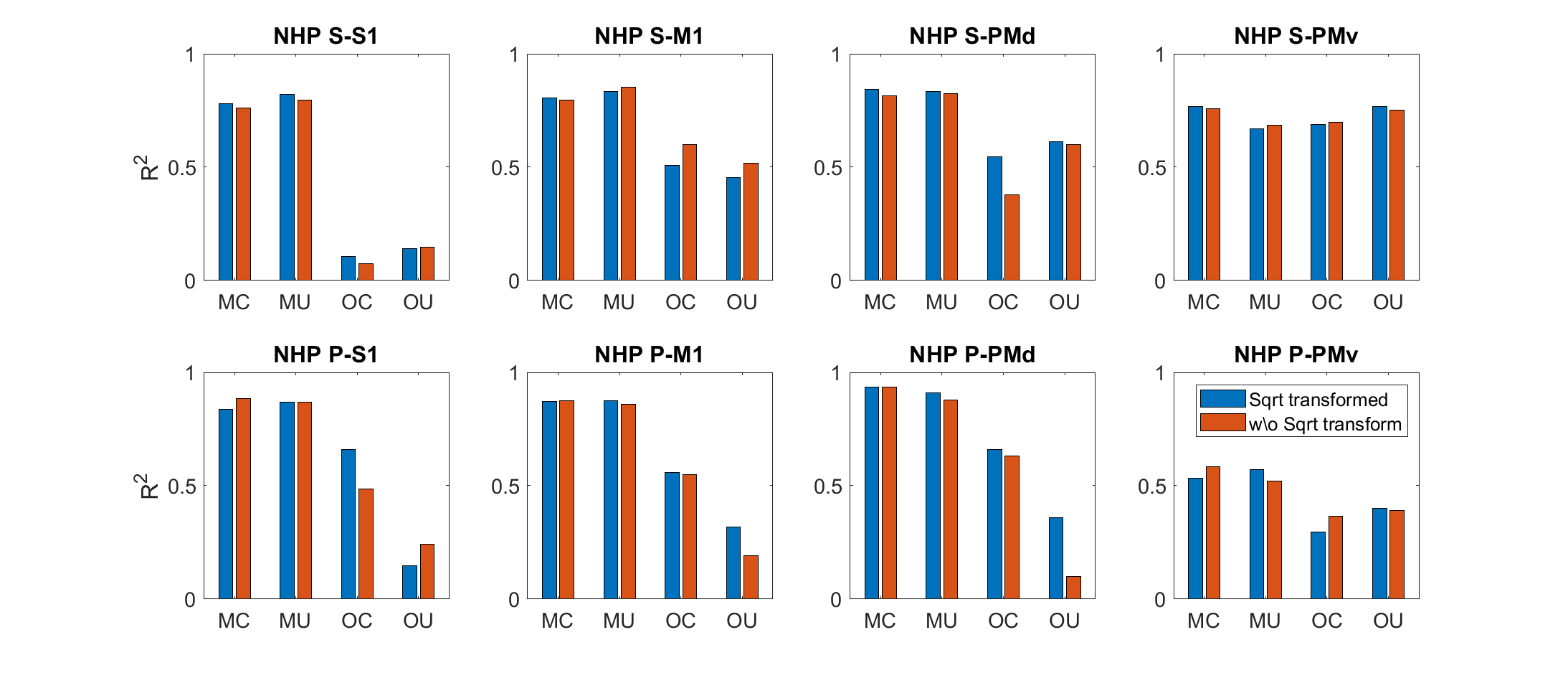


**Figure S4:** The R-square of the decoded grip-force with (blue) or without (red) square root transformation was applied on the spike rate data. The rows of subplots showing data from S1, M1, PMd, and PMv cortices of NHP S and the bottom row are the same for NHP P.

**Algorithm for Linear Regression:** The following algorithm shows the two-step process of collecting a subset of units significant for force to achieve a comparatively better prediction of force using linear regression. **Fig.S5**


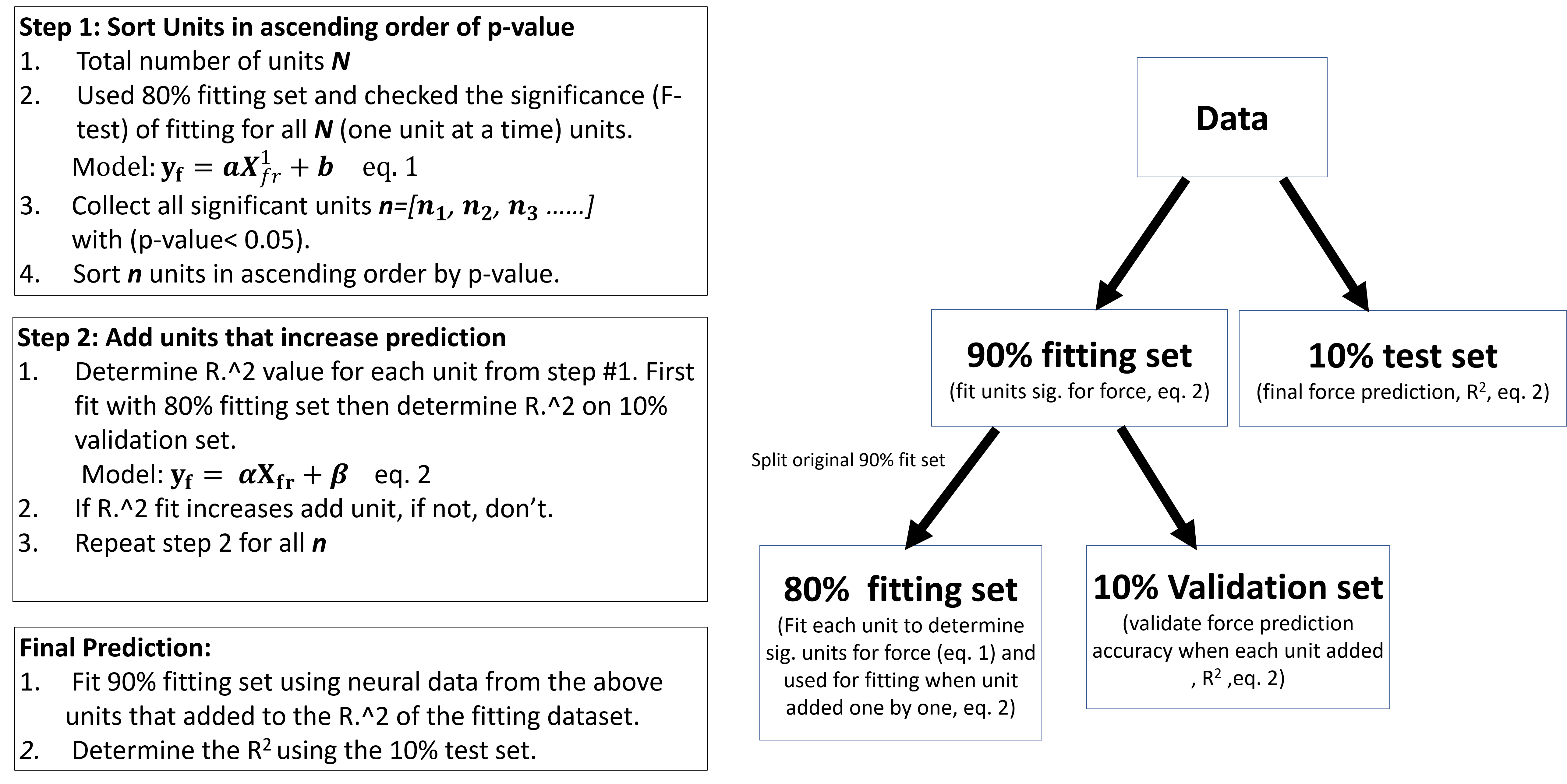


**Figure S5:** Algorithm for linear Regression

**The Force duration for each data block:**

The durations of applied grip-force in R0 (blue) and R1 (red) trials are given on the following box plots for each of the block types (MC= Manual Cued, MU= Manual Uncued, OC= Observation Cued, and OU=Observation Uncued). The box plots show the highest (upper line), 75^th^ percentile, median, 25^th^ percentile, and the lowest values for force duration of all the trials. A paired t-test (p-value <0.05) was performed to check if the R0 peak force was significantly different from the R1 trials. The R0 and R1 force durations are significantly different only for the case of MC block of NHP S.


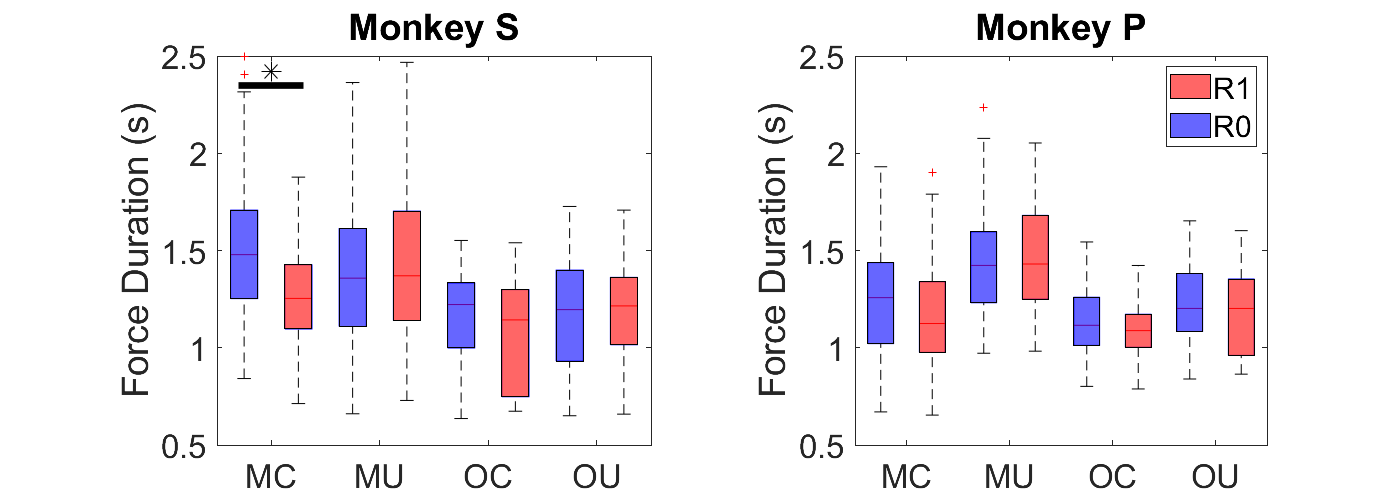


**Figure S6:** The box plot is showing the median, maximum and minimum values with 75^th^ and 25^th^ percentile of the grip-force duration of all the trials on each block individually. The x-axis is showing the data block type (also shown on the legend, which represents the same information). The y-axis is representing the force duration in seconds. The black line and asterisk (*) on the top show the significant difference between R0 (blue) and R1 (red) trials.

**Peak grip-force for each data block:**

The applied/observed peak grip-force on each manual/observational block is plotted on the following figures. The Red represents the R1 trials and blue for R0 trials. The block type (MC, MU, OC, OU) is mentioned in the x-axis. A paired t-test (p-value <0.05) was performed to check if the R0 peak force was significantly different from the R1 trials. The significance test showed no p-value less than 0.05, and hence no asterisk (*) sign was plotted in any of the pairs. The minimum threshold of force for NHPS was 150, and P was 100 for the trial with the lowest force peak threshold. If the value goes below that value, the trial will be failed. The value mentioned is the minimum value for different trails; a random value was added to increase the minimum threshold from 100 (NHP P) and 150 (NHP S).


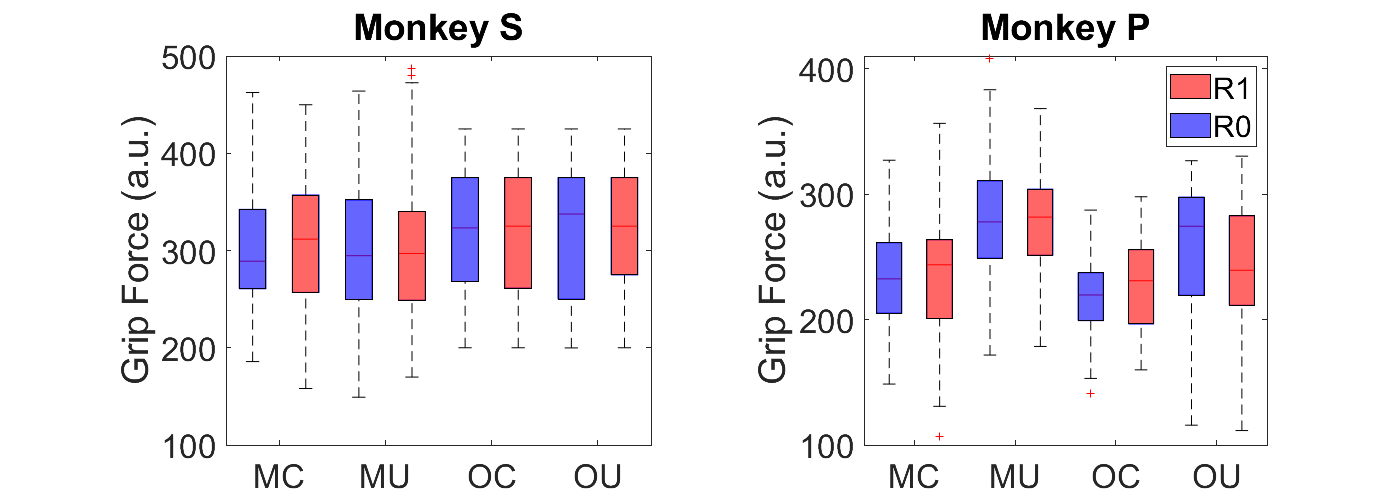


**Figure S7:** The peak force from each block type is plotted on the following box plot, and for each block type, the R0 (blue) and R1 (red) trials are plotted separately for NHP S (left) and NHP P (right). The x-axis is showing the block type (MC, MU, OC, and OU). The y-axis is representing the value of the grip-force applied with arbitrary units. The grip-force values from R0 and R1 trials were significantly different for any case; hence no asterisk (*) is present.

**Reaction time to apply grip-force:**

The reaction time to apply grip-force for R0 and R1 trials is plotted in the following subplots. The manual blocks for NHPS (left) and NHPP (right) are plotted with R0 (blue) and R1 (red). The trials shown are from the same data block (MC, MU). The asterisk (*) on the top of the black line indicates the reaction time is significantly different between R0 and R1 trials (paired t-test, p-value <0.05). The reaction time, in this case, is the difference between the times when the NHP was allowed to apply grip-force (start of the grip-force scene) to the time when they did apply 10% of their peak grip-force on that trial. From the following plot, the reaction time is significantly different only for the manual cued block for NHP S.


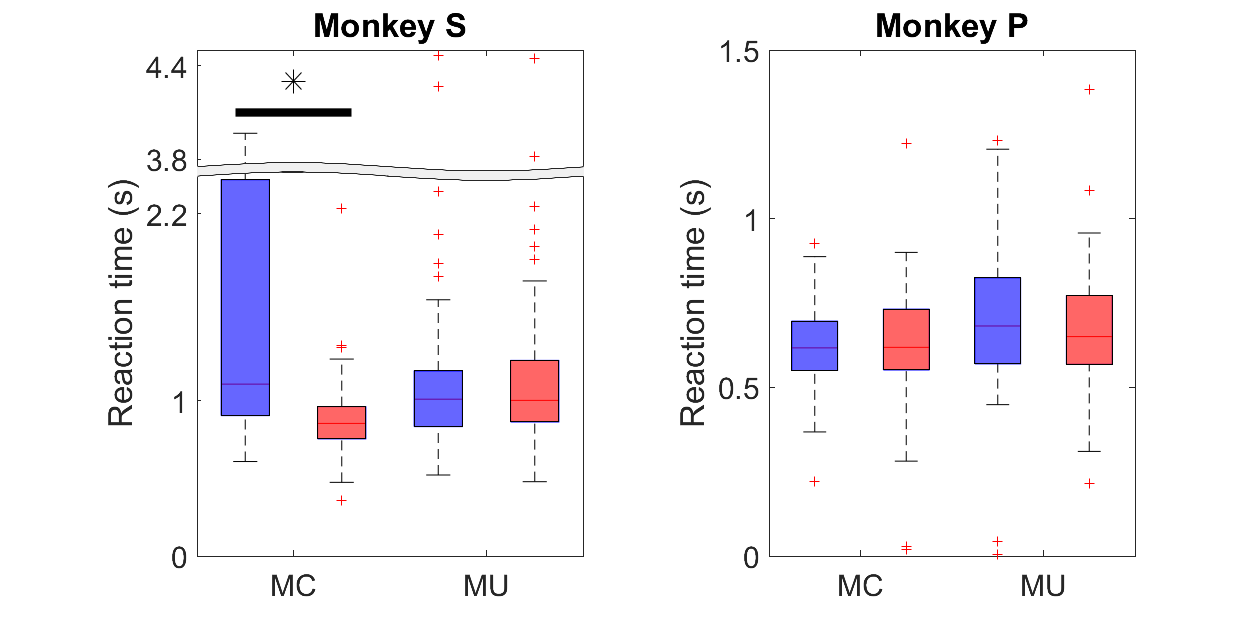


**Figure S8:** The reaction time for R0 (blue) and R1 (red) trials for all the block types is given on the following plots. The x-axis is showing the block types MC, MU for the case of manual blocks. The y-axis is showing the reaction time in seconds. The asterisk (*) on the top of each pair of bar plots represents the reaction time is significantly different between R0 and R1 trials. The left subplot is for NHPS, and the right subplot is for NHPP.

**The number of trials in each data block:**

The following figure shows the number of trials for each data block that was used for analysis.


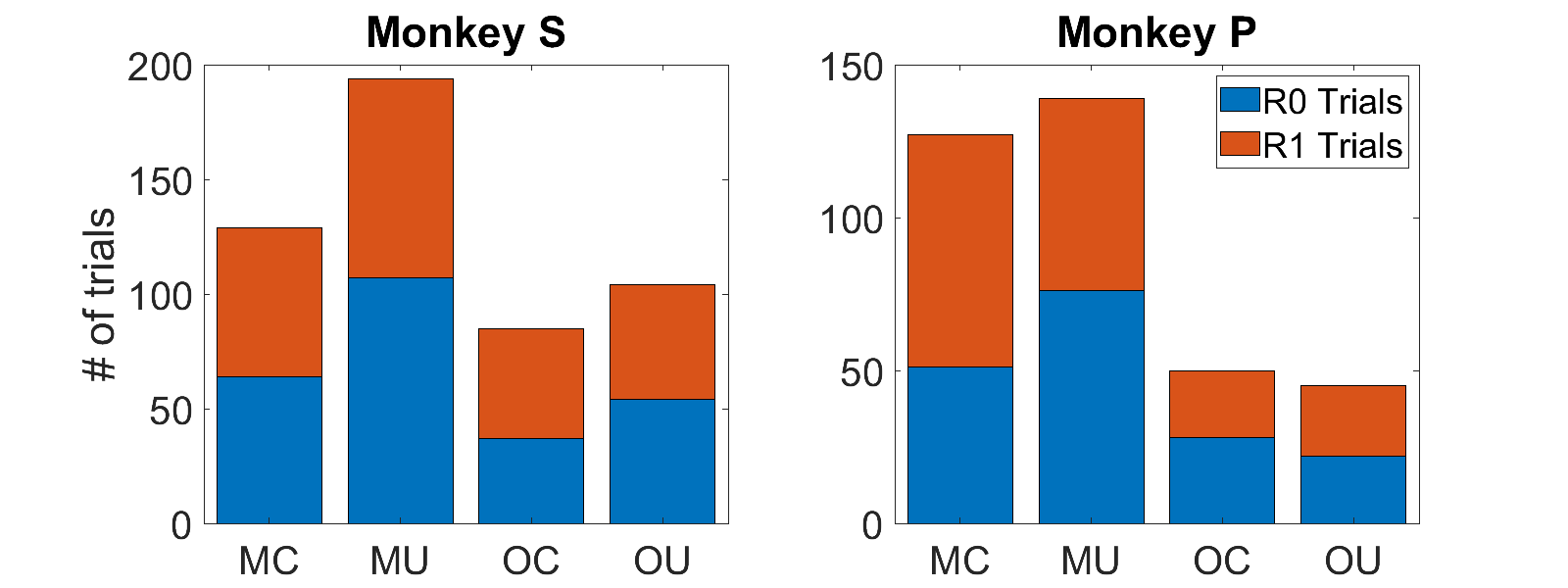


**Figure S9:** The number of trials used for the data analysis is shown in the figure above for NHPS (left) and NHPP (right). Each bar represents the total number of trials, and the blue part of the bar shows R0 trials, where the red part is for R1 trials. The x-axis is representing the block types (MC, MU, OC, and OU), and the y-axis is showing the number of trials.

**Observation Results on force (Fig.S10):**

This figure shows raster plots of some more example units from each of the 4 cortices during grip-force onset and offset taken from observation blocks showing the full average force-time period with both increases and decreases to force onset.


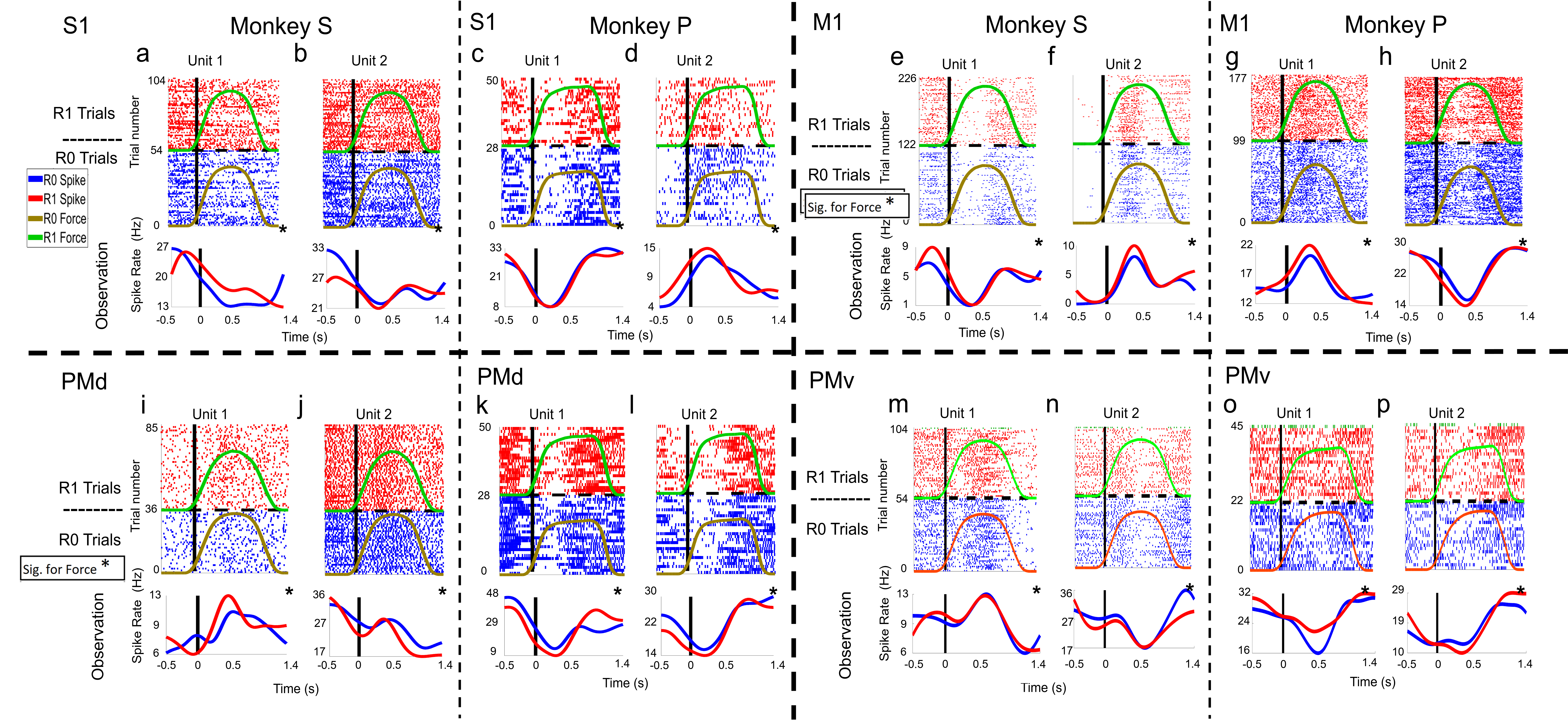


**Figure S10:** Raster plots for units from S1 (plots a, b, c, and d), M1 region (plots e, f, g, and h), PMd (plots i, j, k, and l), and PMv (plots m, n, o and p) are shown for NHPs S and P. These plots show the grip-force values expected given the cued force targets shown to the NHPs during observational blocks. The bold black line on each raster and spike rate plot shows the onset of “force,” and the dotted horizontal line divides R1 (top, red) and R0 (bottom, blue) trials. On each neural data subplot, the x-axis represents time in seconds, and for raster plots, the y-axis represents the trial number (upper part of the subplots), and for the spike rate plot, the y-axis is spike rate in ‘Hz’ (bottom part of the subplots). The Asterisk (*) symbol on the top right corner of the mean spike rate plots represent that unit was significant (F-test, p<0.05) for the force-fit using linear regression model in eq 1.

**Responses of Mirror neuron activity due to grip-force for different data blocks:**

The following figures show the number of MN units in terms of their activity during different blocks. These units are already shown in figure 6 of the main manuscript, but the other category was not elaborated on there, which is given in detail in figures S11 and S12. For analysis, four data blocks for each NHP (NHP S and NHP P) were used. The abbreviations for the block types are MC=Manual Cued, MU=Manual Uncued, OU=Observational Uncued, and OC=Observational Cued. For figure 6, we used the peak value from the absolute correlation coefficients between grip-force and each of the 10 bins of spike rate (100ms from -0.5ms pre grip-force spike rate to 0.5s post, described “Grip-force trajectory prediction” in the methods section) to detect inhibitory or excitatory activity. We considered a unit’s representation excitatory if the value of that peak absolute correlation was positive; otherwise, it was deemed inhibitory.  We detected congruent units among the significant units that showed similar activity in all four data blocks of either inhibitory (blue) or excitatory (red) spike rate in relation to grip-force. Incongruent units showed opposing behavior between manual and observational tasks, such as excitatory during manual and inhibitory during observational, purple, or inhibitory during manual and excitatory during observational (green). Other (gray) units didn’t follow any simple pattern during all four data blocks.

In the following two figures (S11 and S12), we show the “other” units in detail for each combination pattern. The x-axis label is showing four numbers for four data blocks in the order of (MC-MU-OU-OC), and a '0' represents inhibitory activity for that block, and '1' represents excitatory activity. So, for example, the bar with the first label '0000' shows the number of units that showed inhibitory spike rate during grip-force activity during all four data blocks (MC, Mu, OU, and OC). The bar colors are kept as like figure 6 and given as the legend. The bars that contain the other unit category from figure 6 are kept gray-colored here.


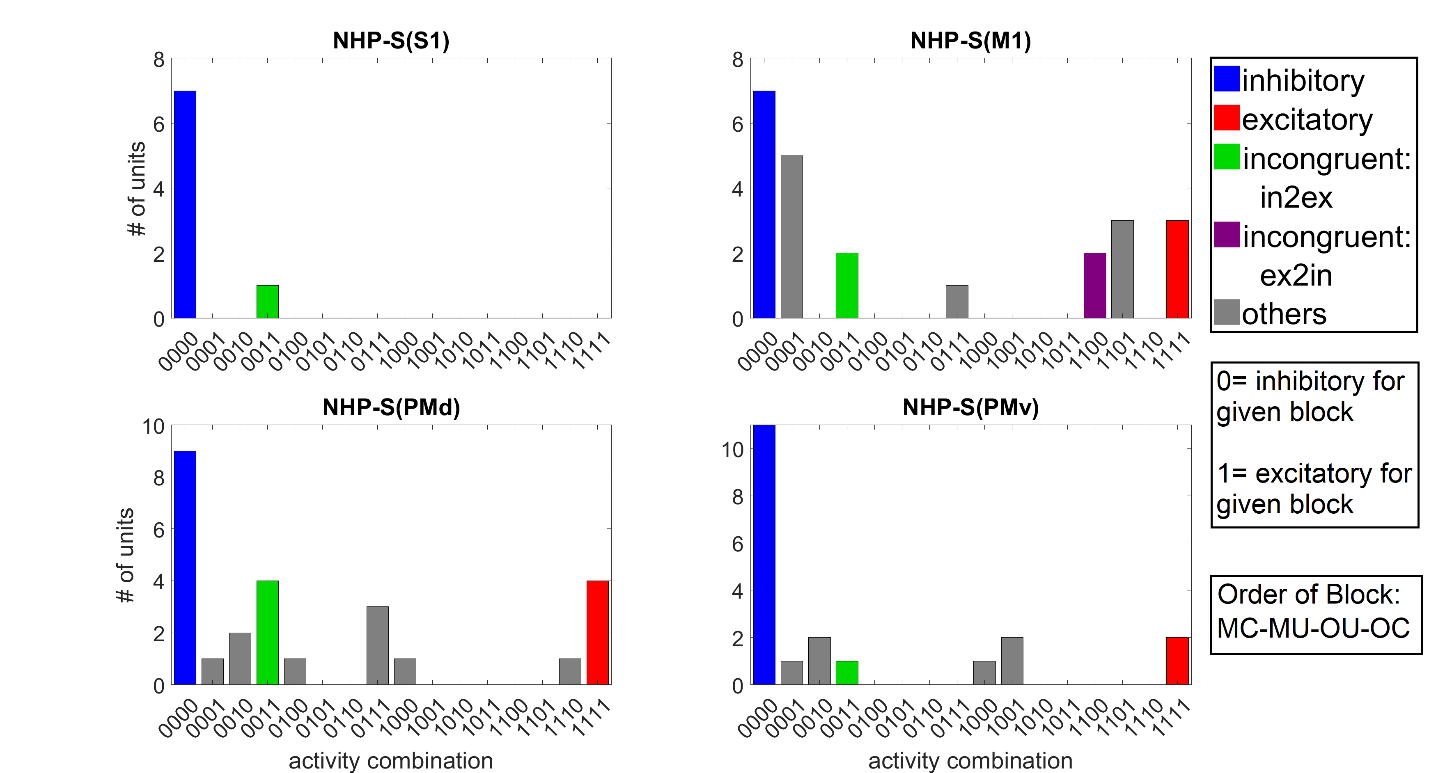

**Figure S11:** The Number of MN units from NHP S for each possible activity combination during blocks in order of MC, MU, OU, and OC. The y-axis represents the number of units, and the x-axis label shows the combination of inhibitory (0) or excitatory activity for the given four data blocks. The title on each subplot indicates the corresponding cortex and NHP name.


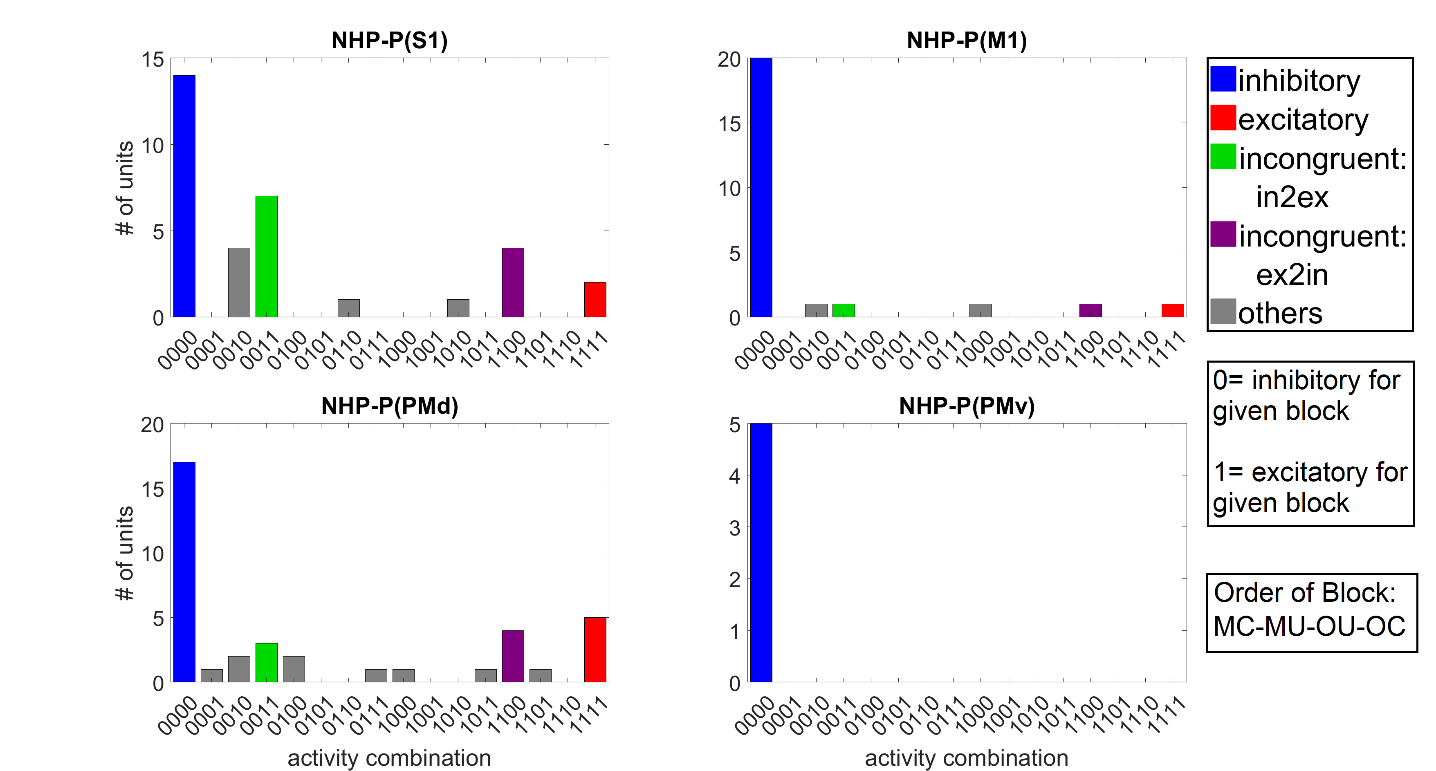


**Figure S12:** The Number of MNs from NHP P for each possible activity combination during blocks in order of MC, MU, OU, and OC. The y-axis represents the number of units, and the x-axis label shows the combination of inhibitory (0) or excitatory activity for the given four data blocks. The title on each subplot indicates the corresponding cortex and NHP name.

**Neural Time Lag Analysis (Fig.S13):**


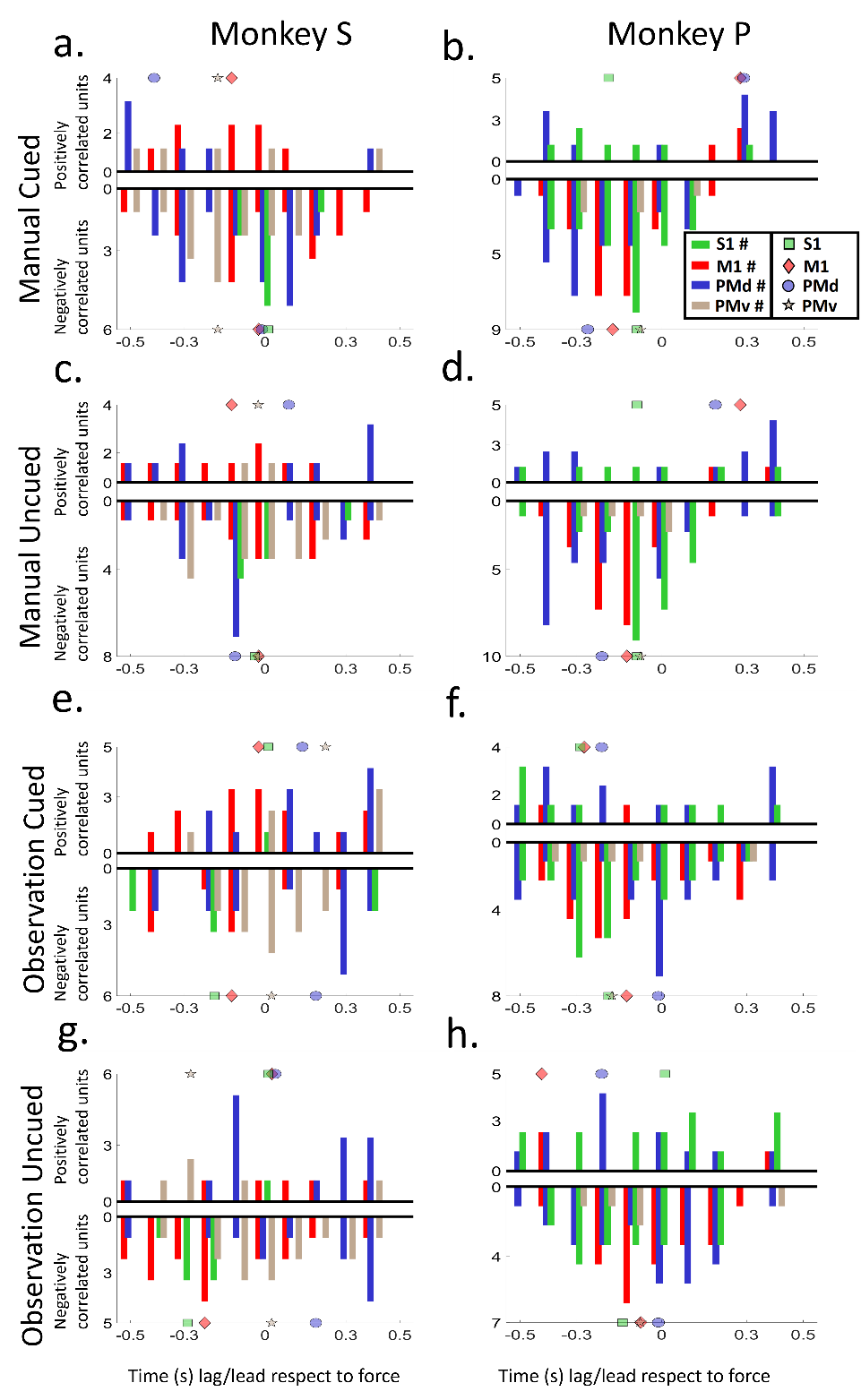
The time-dependent peak correlation plots for all the blocks are given below. Only units significant for force encoding that were tracked through all manual and observational blocks are shown below.

**Figure S13**: The number of units that had their highest correlation between force and spike rate for each time bin are shown leading (0.5s before) or lagging (0.5s after) each force value (100ms non-overlapping time bins). Units significant (F-test, p <0.05) on all block types were plotted in the time bin where their correlation coefficient with force was maximum in absolute value, either positive or negative. The y-axis represents the number of units (positively correlated units on the upper side and negatively correlated units on the lower). The x-axis is the time lag/lead for the maximum correlation bin. Units from S1 (a, b), M1 (c, d), PMd (e, f), and PMv (g, h) cortices are given in each plot.

**Distribution of Significant Correlation Time Lags Between Neural Activity and Force (Fig.S14):**

The following figure shows the time shift of significant peak correlation from manual cued blocks to manual uncued blocks and from observation cued blocks to observation uncued blocks. See Fig.7 in the main text for more of such plots between manual and observational.


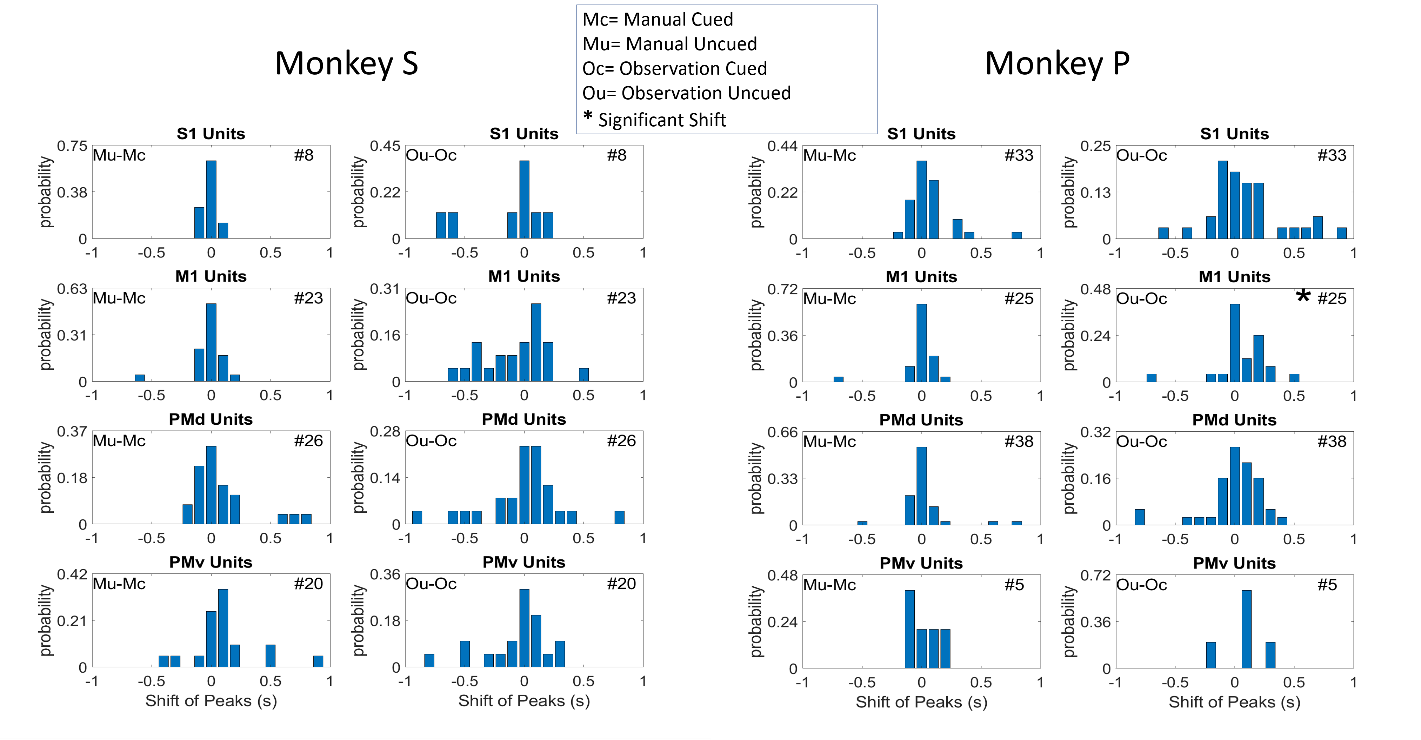


**Figure S14**: The shift of peak force correlation from manual to manual and observational to observational block. Bar plots showing the shift of peak correlation between Manual blocks and Observational blocks for S1, M1, PMd, and PMv cortex for individual MNs. The change from a block type to another block type is given on the left of each figure, and the abbreviations of the block types are (Mc= Manual Cued, Mu= Manual Uncued, Oc= Observation Cued, Ou= Observation Uncued). The number of units used is included in the upper right corner of each subplot. An asterisk (*) before the number of units represents a significant shift in histograms between the blocks used for that subplot. The shift was calculated by subtracting cued manual histogram from the uncued Manual histogram and similarly for the observational cases.

**Spike Rate vs. Force** (Manual)


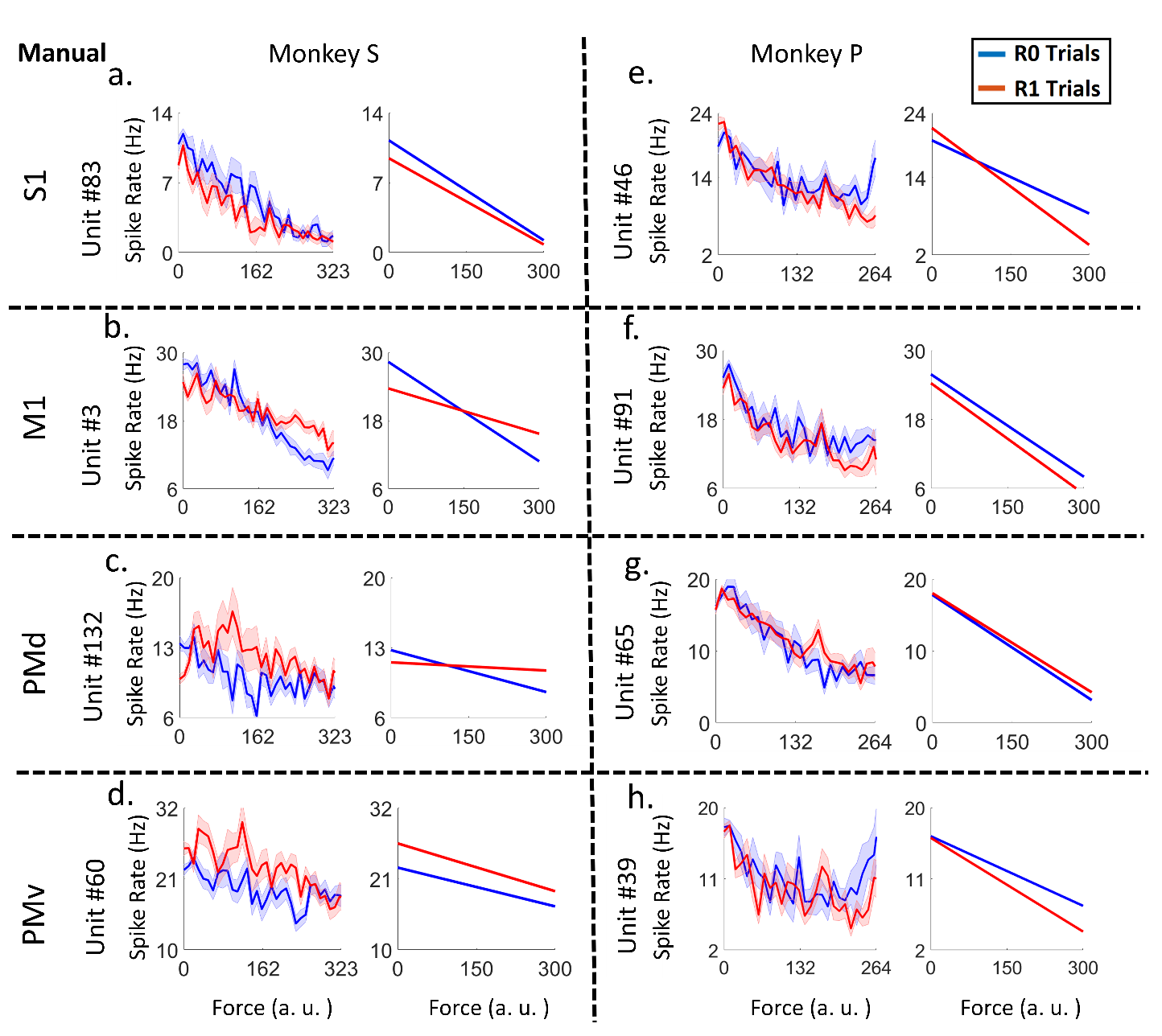


**Figure. S15**: Plots of spike rate vs. force (left subplots) and their linear tuning curves (right subplots) for Grip-force manual trials as compared to Fig.9 in the main text for the observational. The subplots show example units from S1 (plot *a, e*), M1 (plot *b, f*), PMd (plot *c, g*), and PMv (plot *d, h*) cortices of both NHPs (for NHP S plots *a, b, c,* and *d* and for NHP P plots *e, f, g,* and *h*). The units presented here showed a significant difference between R0 and R1 trials (ANCOVA, F-test, p<0.05) for force tuning curves during manual trials. Red lines indicate rewarding trials (R1), and blue indicate non-rewarding trials (R0).

**Regression on additional observational blocks:**

Table S1 shows the regression analysis for NHP S and P for two addition observational blocks recorded a different day than used for the main text plots. The table contains the R-square values for model fits given in equation 1 and the corresponding p-values and F-statistics. The R-squared values for prediction and their p-values are shown in the table as well.

**Table S1**: Regression Model Output for NHP S & P for the two additional observational blocks recorded on a different day with units we could not track from the previous data sets.

| NHP S - S1 | NHP P – S1 |
| --- | --- |
| \| Block \| R-fit \| p-value \| F-stat \| R-predict \| p-value \| \| --- \| --- \| --- \| --- \| --- \| --- \| \| Observation  Cued \| 0.28 \| 3e-91 \| 3.81 \| 0.19 \| 8.3e-21 \| \| Observation  Uncued \| 0.34 \| 4.3e-154 \| 4.79 \| 0.04 \| 9.3e-6 \| | \| Block \| R-fit \| p-value \| F-stat \| R-predict \| p-value \| \| --- \| --- \| --- \| --- \| --- \| --- \| \| Observation  Cued \| 0.46 \| 1.3e-229 \| 8.34 \| 0.27 \| 1.9e-26 \| \| Observation  Uncued \| 0.53 \| 0 \| 10.09 \| 0.13 \| 1.8e-14 \| |
| NHP S – M1 | NHP P – M1 |
| \| Block \| R-fit \| p-value \| F-stat \| R-predict \| p-value \| \| --- \| --- \| --- \| --- \| --- \| --- \| \| Observation  Cued \| 0.65 \| 0 \| 11.59 \| 0.6 \| 2.6e-87 \| \| Observation  Uncued \| 0.76 \| 0 \| 21.19 \| 0.66 \| 4.3e-113 \| | \| Block \| R-fit \| p-value \| F-stat \| R-predict \| p-value \| \| --- \| --- \| --- \| --- \| --- \| --- \| \| Observation  Cued \| 0.61 \| 0 \| 13.19 \| 0.55 \| 2.7e-64 \| \| Observation  Uncued \| 0.69 \| 0 \| 18.07 \| 0.55 \| 3.1e-75 \| |
| NHP S – PMd | NHP P – PMd |
| \| Block \| R-fit \| p-value \| F-stat \| R-predict \| p-value \| \| --- \| --- \| --- \| --- \| --- \| --- \| \| Observation  Cued \| 0.53 \| 1.9e-273 \| 6.63 \| 0.36 \| 2.7e-43 \| \| Observation  Uncued \| 0.67 \| 0 \| 11.57 \| 0.39 \| 0.55 \| | \| Block \| R-fit \| p-value \| F-stat \| R-predict \| p-value \| \| --- \| --- \| --- \| --- \| --- \| --- \| \| Observation  Cued \| 0.5 \| 6.4e-274 \| 10 \| 0.52 \| 6.3e-59 \| \| Observation  Uncued \| 0.55 \| 0 \| 13.14 \| 0.37 \| 2e-44 \| |
| NHP S – PMv | NHP P – PMv |
| \| Block \| R-fit \| p-value \| F-stat \| R-predict \| p-value \| \| --- \| --- \| --- \| --- \| --- \| --- \| \| Observation  Cued \| 0.6 \| 0 \| 11.84 \| 0.72 \| 5.7e-121 \| \| Observation  Uncued \| 0.8 \| 0 \| 34.11 \| 0.72 \| 6.1e-134 \| | \| Block \| R-fit \| p-value \| F-stat \| R-predict \| p-value \| \| --- \| --- \| --- \| --- \| --- \| --- \| \| Observation  Cued \| 0.35 \| 3.5e-197 \| 11.24 \| 0.28 \| 4.9e-27 \| \| Observation  Uncued \| 0.46 \| 0 \| 13.18 \| 0.36 \| 1.4e-42 \| |

**Table S2: Regression Model Output for NHPs S & P for Fig.5 in the Main Text**

| NHP S - S1 | NHP P – S1 |
| --- | --- |
| \| Block \| R^2^-fit \| p-value \| F-stat \| R^2^-predict \| p-value \| \| --- \| --- \| --- \| --- \| --- \| --- \| \| Manual  Cued \| 0.91 \| 0 \| F_550,2173_  39.83 \| 0.78 \| 1.9e-101 \| \| Manual Uncued \| 0.89 \| 0 \| F_540,3597_  51.68 \| 0.82 \| 3.5e-173 \| \| **Obs**  **Cued** \| 0.34 \| 7.4e-40 \| F_250,1330_  3.32 \| **0.11** \| 9.5e-6 \| \| **Obs**  **Uncued** \| 0.29 \| 9.4e-46 \| F_300,1679_  3.47 \| **0.14** \| 7.8e-9 \| | \| Block \| R^2^-fit \| p-value \| F-stat \| R^2^-predict \| p-value \| \| --- \| --- \| --- \| --- \| --- \| --- \| \| Manual  Cued \| 0.78 \| 0 \| F_320,2142_ 24.19 \| 0.84 \| 2.26e-109 \| \| Manual  Uncued \| 0.9 \| 0 \| F_470,2528_ 46.77 \| 0.87 \| 4.6e-148 \| \| **Obs**  **Cued** \| 0.68 \| 5e-88 \| F_220,707_  7.12 \| **0.66** \| 1.4e-25 \| \| **Obs**  **Uncued** \| 0.47 \| 2.3e-42 \| F_220,652_  4.6 \| **0.15** \| 9.5e-5 \| |
| NHP S – M1 | NHP P – M1 |
| \| Block \| R^2^-fit \| p-value \| F-stat \| R^2^-predict \| p-value \| \| --- \| --- \| --- \| --- \| --- \| --- \| \| Manual  Cued \| 0.92 \| 0 \| F_590,2133_44.24 \| 0.80 \| 7.8e-109 \| \| Manual  Uncued \| 0.93 \| 0 \| F_530,3607_85.7 \| 0.83 \| 1.2e-180 \| \| **Obs**  **Cued** \| 0.69 \| 1.7e-147 \| F_240,1340_6.91 \| **0.51** \| 1.6e-28 \| \| **Obs**  **Uncued** \| 0.65 \| 1.2e-197 \| F_350,1629_8.19 \| **0.45** \| 1.8e-30 \| | \| Block \| R^2^-fit \| p-value \| F-stat \| R^2^-predict \| p-value \| \| --- \| --- \| --- \| --- \| --- \| --- \| \| Manual  -Cued \| 0.80 \| 0 \| F_360,2102_=23.71 \| 0.87 \| 5.58e-123 \| \| Manual  -Uncued \| 0.89 \| 0 \| F_440,2558_46.22 \| 0.87 \| 1.6e-151 \| \| **Obs**  **Cued** \| 0.76 \| 6.3e-96 \| F_300,627_ 7.29 \| **0.56** \| 7e-20 \| \| **Obs**  **Uncued** \| 0.57 \| 4.5e-52 \| F_180,692_ 4.81 \| **0.32** \| 1.5e-09 \| |
| NHP S –PMd | NHP P – PMd |
| \| Block \| R^2^-fit \| p-value \| F-stat \| R^2^-predict \| p-value \| \| --- \| --- \| --- \| --- \| --- \| --- \| \| Manual  Cued \| 0.92 \| 0 \| F_550,2173_44.25 \| 0.84 \| 1.3e-122 \| \| Manual  Uncued \| 0.92 \| 0 \| F_640,3497_58.86 \| 0.83 \| 9.1e-181 \| \| **Obs**  **Cued** \| 0.66 \| 6.3e-172 \| F_270,1310_8.99 \| **0.54** \| 1.5e-31 \| \| **Obs**  **Uncued** \| 0.73 \| 2.5e-282 \| F_330,1649_11.95 \| **0.61** \| 6.4e-47 \| | \| Block \| R^2^-fit \| p-value \| F-stat \| R^2^-predict \| p-value \| \| --- \| --- \| --- \| --- \| --- \| --- \| \| Manual  Cued \| 0.85 \| 0 \| F_260,2202_47.17 \| 0.94 \| 4.9e-164 \| \| Manual  Uncued \| 0.93 \| 0 \| F_540,2458_63.09 \| 0.91 \| 6.4e-175 \| \| **Obs**  **Cued** \| 0.73 \| 1.3e-117 \| F_240,627_ 9.79 \| **0.66** \| 1.4e-25 \| \| **Obs**  **Uncued** \| 0.72 \| 4.7e-82 \| F_200,672_ 6.71 \| **0.36** \| 7.3e-11 \| |
| NHP S –PMv | NHP P – PMv |
| \| Block \| R^2^-fit \| p-value \| F-stat \| R^2^-predict \| p-value \| \| --- \| --- \| --- \| --- \| --- \| --- \| \| Manual  Cued \| 0.83 \| 0 \| F_430,2293_26.72 \| 0.77 \| 2.3e-97 \| \| Manual  Uncued \| 0.87 \| 0 \| F_610,3527_40.29 \| 0.67 \| 4.4e-112 \| \| **Obs**  **Cued** \| 0.81 \| 4e-265 \| F_320,1260_13.65 \| **0.69** \| 4.9e-46 \| \| **Obs**  **Uncued** \| 0.86 \| 0 \| F_430,1549_16.25 \| **0.77** \| 7.1e-71 \| | \| Block \| R^2^-fit \| p-value \| F-stat \| R^2^-predict \| p-value \| \| --- \| --- \| --- \| --- \| --- \| --- \| \| Manual  Cued \| 0.61 \| 5.3e-308 \| F_24,2222_ 14.26 \| 0.53 \| 9.3e-47 \| \| Manual  Uncued \| 0.6 \| 0 \| F_160,2838_26.31 \| 0.57 \| 5.6e-63 \| \| **Obs**  **Cued** \| 0.43 \| 1.2e-60 \| F_80,847_ 7.89 \| **0.29** \| 2.7e-09 \| \| **Obs**  **Uncued** \| 0.33 \| 7.9e-45 \| F_80,792_ 8.15 \| **0.40** \| 2.9e-12 \| |

**Table S2:** Results from the regression analysis for NHPs S and P between neural activity and force trajectories. The table contains the R-square value for the model (eq. 2) fit and prediction, including their corresponding p-values and F-statistics. We have used bold text for the prediction during observation tasks. Note all single units were followed through each of the 4 task types on a given day. From table 1, it appears that these brain regions represent force during both the manual trials and the observational versions of these trials. In Fig.6 we show the number of single units that significantly predict force during both manual and observational trails, indicating that these same units represent this information to some extent during both manual and observed movements, which may represent a new form of mirror neuron activity.

**Table S3**: Units with significantly different slopes for the force tuning curves between R0 and R1. The values in parenthesis show the adjusted significant units after Benjamini and Hochberg’s method accounting for the false discovery rate was applied. The BH method applied for the number of units on which the hypothesis testing was applied in the population is described in the method section titled “grip-force tuning curve analysis”.

|  | S1 # | S1 % | M1 # | M1 % | PMd # | PMd % | PMv # | PMv % |
| --- | --- | --- | --- | --- | --- | --- | --- | --- |
| NHP S Manual | 11 (3) | 8 (2) | 20 (4) | 14 (3) | 28 (24) | 20 (17) | 13 (4) | 8 (2) |
| NHP P Manual | 9 (1) | 7 (1) | 9 (4) | 8 (3) | 16 (6) | 14 (5) | 4 (0) | 5 (0) |
| NHP S Obs. | 2 (2) | 2 (2) | 2 (1) | 2 (1) | 2 (0) | 2 (0) | 0 (0) | 0 (0) |
| NHP P Obs. | 0 (0) | 0 (0) | 1 (0) | 1 (0) | 2 (0) | 2 (0) | 1 (1) | 2 (2) |

**Table S4**: Units with significantly different y-intercept for the force tuning curves for R0 and R1. The values in parenthesis show the adjusted significant units after Benjamini and Hochberg’s method accounting for the false discovery rate was applied. The BH method applied for the number in the population is described in the method section titled “grip-force tuning curve analysis”.

|  | S1 # | S1 % | M1 # | M1 % | PMd # | PMd % | PMv # | PMv % |
| --- | --- | --- | --- | --- | --- | --- | --- | --- |
| NHP S Manual | 14 (2) | 10 (1) | 29 (21) | 21 (15) | 32 (24) | 23 (17) | 27 (17) | 16 (10) |
| NHP P Manual | 8 (0) | 6 (0) | 7 (1) | 6 (1) | 14 (4) | 12 (3) | 2 (0) | 2 (0) |
| NHP S Obs. | 3 (3) | 3 (3) | 4 (4) | 3 (3) | 1 (1) | 1 (1) | 4 (4) | 3 (3) |
| NHP P Obs. | 0 (0) | 1 (0) | 1 (0) | 1 (0) | 1 (0) | 1 (0) | 2 (0) | 3 (0) |

**EMG Analysis**

The data used in the main text had corrupted EMG, and we were unable to confirm whether the NHPs were still using their arm muscles during the observation task; however, from observation notes and the evident decrease in activity seen in Figs 3-4 of the main text, and the fact that the NHPs could not reach for, or touch the force transducing handle, we do not believe the NHPs were physically rehearsing grip movements during observation. In addition, as seen below in Fig.S16, EMG on other days from these same NHPs was available during observation trials and did not show any obvious activation patterns in the mean activity; however, it appears there was some small and insignificant increase in biceps for NHP P. The neural results of these below data sets held to that shown in the main text and figs. This does not rule out the NHPs covertly imagining movement.


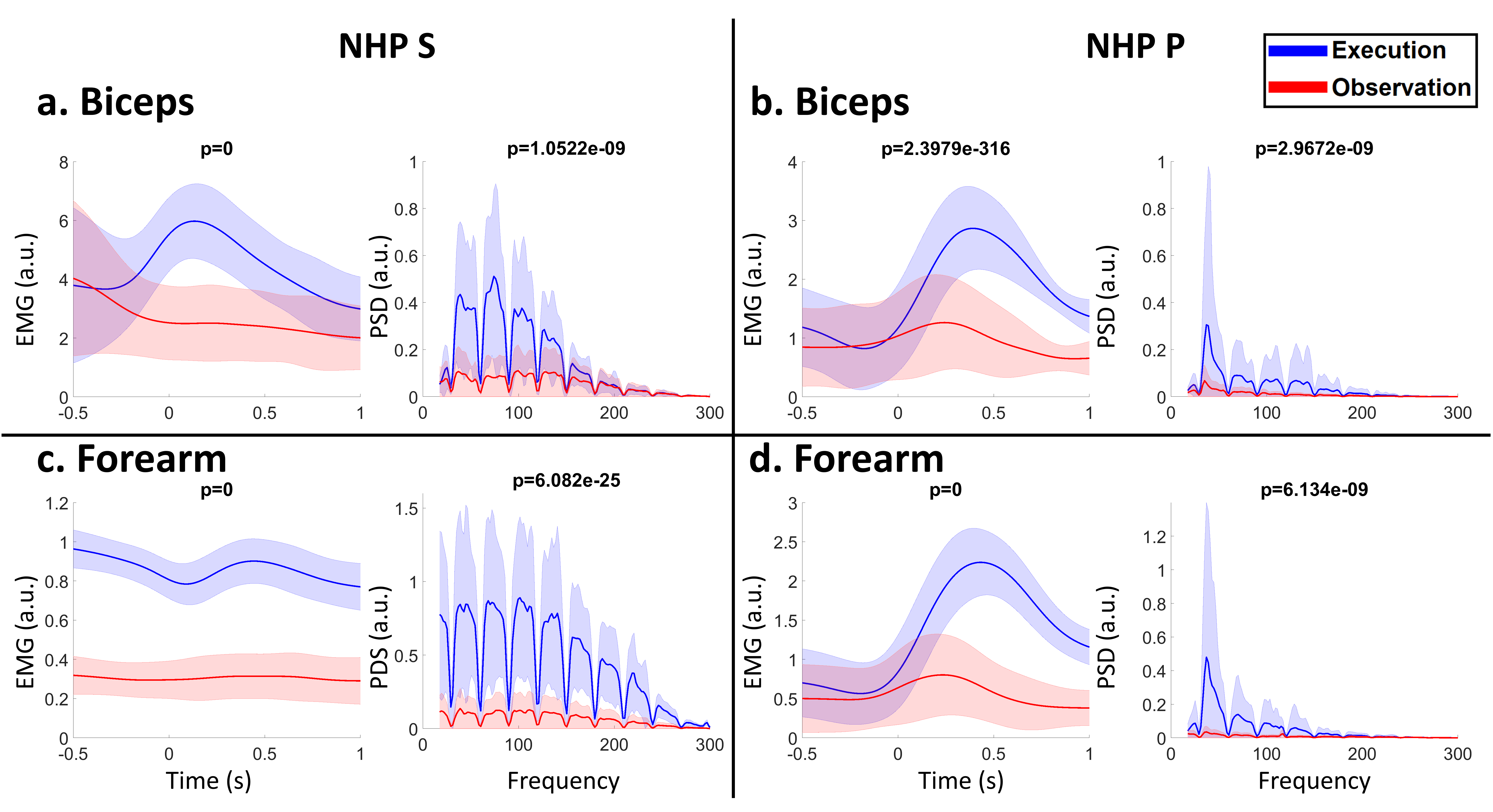


**Figure S16**: EMG collected from the Biceps (section a and b) and Forearm (section c and d) of NHP S (section a and c) and P (section b and d) recorded on different days from that used in the main text. The blue color represents manual blocks (execution), and red is for observation. On each of four sections, a, b, c, and d, left plots to show the mean EMG (0.5s pre-force onset to 1s post force onset activity) for all the trials with standard deviation as shaded error bar. The right plots are showing the mean power spectral density (PSD) with standard deviation for the same data. A bandpass filter was applied on the EMG for the power line noise and its harmonics (30Hz, 60Hz, 90Hz… etc.), and the effect of that can be seen in the PSD plots (b). The p-value for the significance test between the mean manual and observational is given on the title of each subplot.

**Units significant for Extensor Muscle EMG:**

Figure S13 shows, most recorded units are showing a negative correlation with grip-force, and we speculated that it could be that they are connected to the extensor muscle groups in the forearm. To investigate further, we determined if units showing a negative correlation with force would have a positive correlation with the EMG recorded from the extensor muscle groups. We used manual data blocks recorded on another day for NHP S, which had EMG recorded for the extensor muscle from the forearm. Unfortunately, NHP P extensor EMG was not recorded on the other days. For correlation, EMG and spike data were taken from all trials and concatenated. We used binned (100ms) spike data taken from 500ms pre-force onset to 1000ms post and measured the spearman rank correlation with the corresponding binned envelope of the EMG data from the extensor muscles of the forearm. The units that showed significant force-fit and a negative correlation with the grip-force were further investigated to observe their correlation with extensor muscle EMG with the procedure described above. The percentage of such units that have a positive correlation with extensor muscle EMG and a negative correlation with grip-force for NHP S is shown in figure S17. Figure S18 is showing the mean EMG and spike rate for an example unit with the raster plot.


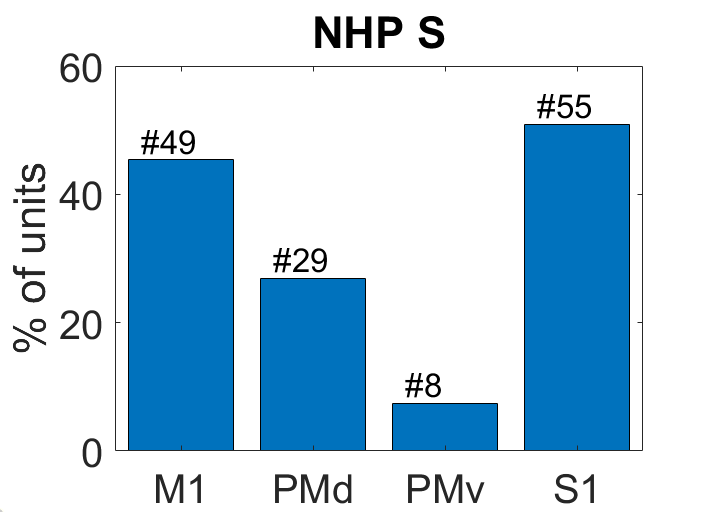


**Figure S17:** Percentage of MNs with a positive correlation between spike activity and extensor EMG envelope from the units that showed negative correlation with grip-force.


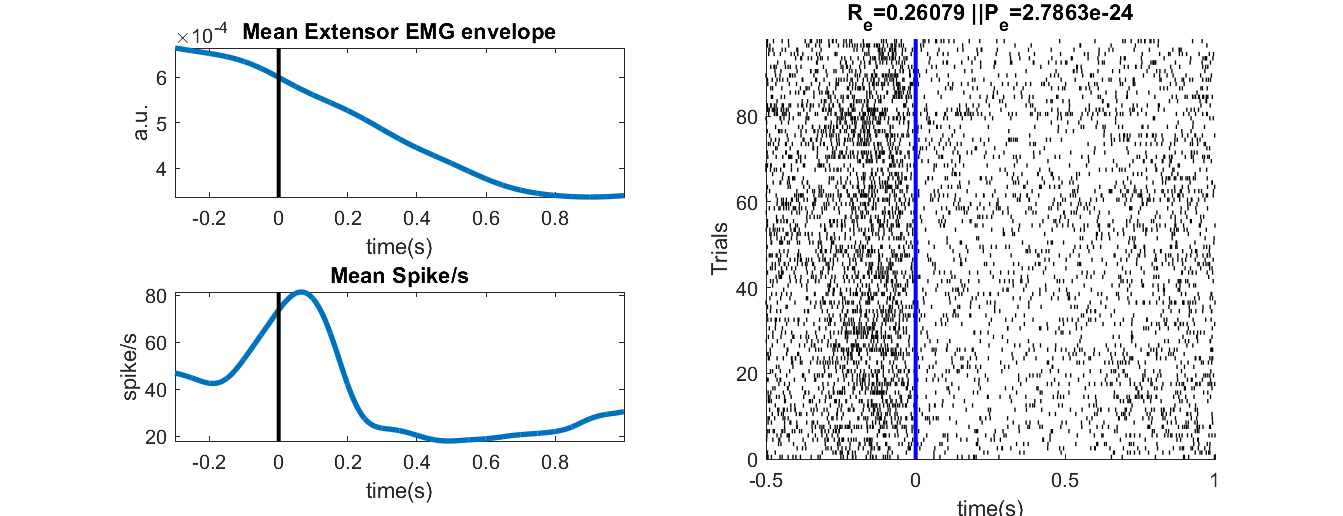


**Figure S18:** An example unit taken from NHP S M1 cortex that shows a positive correlation with the Extensor EMG activity. The left plots show the mean EMG envelope (top) and the mean spike activity (bottom) for all trials. The right raster plot shows spike information for all trials. The title on the raster plot is showing the Spearman rank correlation (Re) with the significance (Pe) between binned (100ms) spike rate and extensor EMG done on concatenated data from all trials.

**Significant units for Peak Grip-force:**

As force profiles were stereotypical smooth Gaussian-like profiles, we wanted to make sure our regression model fits and predictions were meaningful and not solely due to a unit having a phasic response that could be used to fit the stereotypical waveform. Therefore, we determined how neural activity correlated with the peak values of the Gaussian-shaped force profiles during each trial. We considered force values around the peak force applied on each trial (three values around the peak including the peak value), and we placed neural data, centered around each of them, into ten 100ms non-overlapping bins from 500ms pre-peak force to 500ms post-peak force neural activity. Each MN (figure 6 on the main manuscript) was tested for peak force significance (F-test, BH corrected for the number of corresponding units) by fitting a linear regression model with peak force values and spike rate for each of the ten bins. The blue bars are showing the units with Force-Mirror-neuron properties (Figure 6 inside the main manuscript). The red bars in figure S19 show significant units for peak grip-force activity among the force-mirror-neuron units.


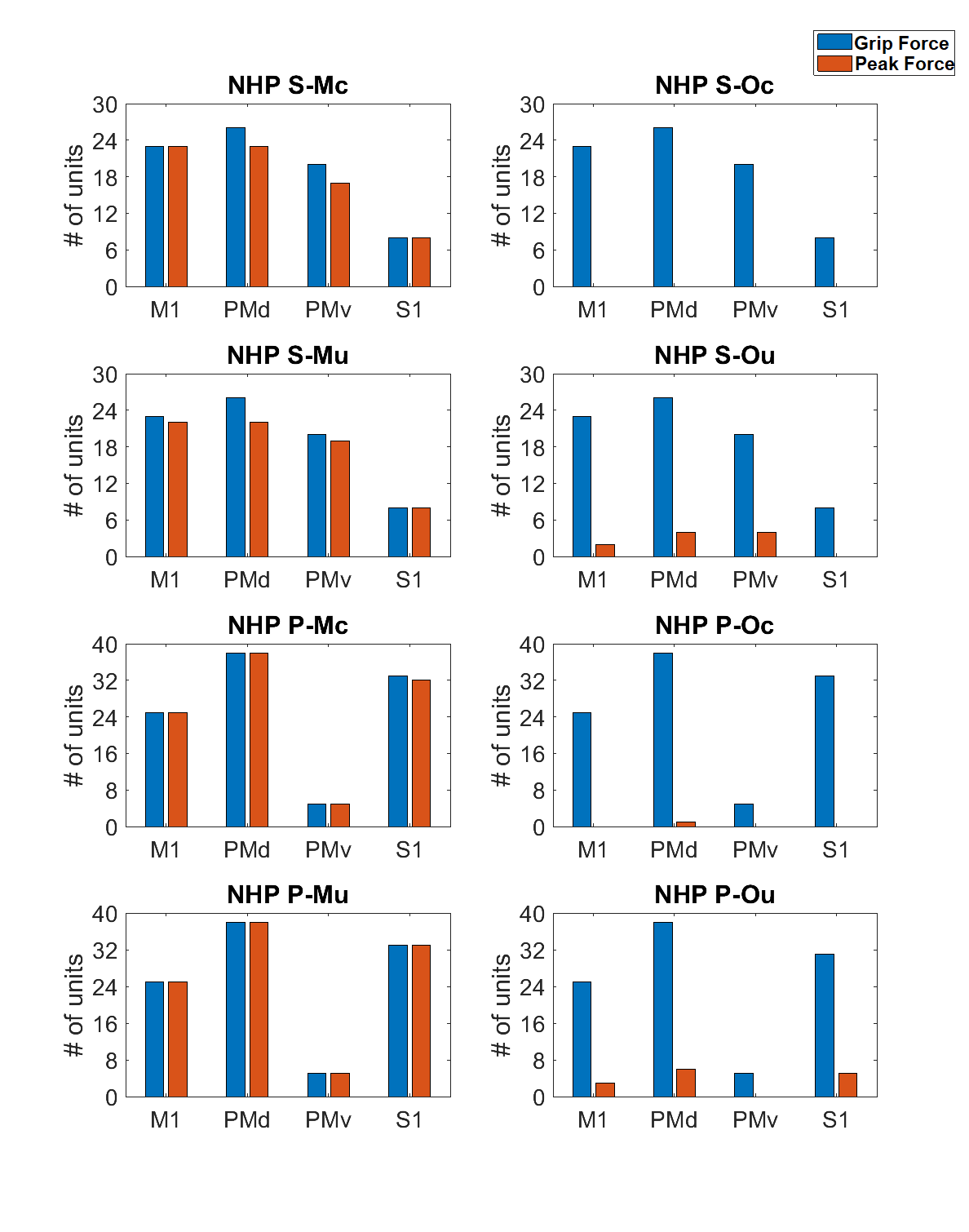


**Figure S19:** This plot shows the MN information from Figure 6 of the main manuscript. The blue bars represent units significant for grip-force (F-test, BH corrected for the number of units in the population), and red bars represent units significant for peak grip-force values (F-test, BH corrected for the number of units in the population). The abbreviations on the subplot titles have the following meanings: Mc = Manual cued block, Oc = Observation cued block, Mu = Manual uncued block, and Ou = Observation uncued block.

**Behavioral Summary:**

Two NHP subjects participated in our experiment, one male *Macaca* Radiata (NHP S) and one female *Macaca* Mulatta (NHP P). Both NHPs performed manual and observational tasks. During the observational version of the task, a plexiglass box restrained their hand from reaching the grip-force sensor. For the manual version of the task, they were able to reach the force sensor. During manual trials, when they were cued to apply grip-force, they reached to the grip-force sensor, and at the end of a trial, they usually rested their arm on the base of the grip-force sensor. NHP S achieved success rates of 77% (cued manual) and 82% (uncued manual) during the two manual-task blocks, while NHP P achieved rates of 58% (cued manual) and 72% (uncued manual). This comparatively low success rate for the manual block when they were cued for the reward at the beginning shows that both NHPs had an idea about the reward information. Between the two monkeys, the performance of NHP S during the trial was affected more by reward cue information than NHP P. Figure S6 shows the force duration is significantly different between R0 and R1 trials for NHP S, which is not the same for NHP P. Also, from figure S7 we can see that the reaction time is significantly longer for NHP S during R0 trials than R1 trials. Both results are an indication that NHP S task performance was more dependent on reward compared to NHP P. One thing that should be mentioned is that, for NHP S, although reaction time and grip-force duration were significantly different the peak force applied on each trial was not significantly different (figure S11) since the applied grip-force was supposed to be within a boundary value cued during the task.

**Directionality of the Mirror Neuron Distribution About the array center:**

The positions of the mirror neurons with respect to the electrodes they were recorded from is shown in Fig.2 in the main manuscript. To observe any non-uniform distribution of MNs with respect to the center of the array, we applied the Rayleigh test, which used the angular position of all the MNs from the center of the array and tested the hypothesis that the angles of the MNs are uniformly distributed. For each cortex, we applied the test for both NHPs individually, and we also combined data from both NHPs for each cortex and applied the statistics again. Table S5 shows the p-value recorded from the statistical tests. Only the M1 cortex showed significance for all cases with a mean direction toward (Fig. 2, Main text) pre-motor cortex.

**Table S5**: the p-value from the Rayleigh test.

|  | S1 | M1 | PMd | PMv |
| --- | --- | --- | --- | --- |
| NHP S | 0.234 | 0.0036 | 0.7366 | 0.0595 |
| NHP P | 0.4617 | 0.0045 | 0.4104 |  |
| Combined | 0.5714 | 1.48E-05 | 0.3726 |  |

**Pruned Data Analysis:**

As seen in Fig.S6 NHP S had a significant difference in force duration between the R0 and R1 trials. We wanted to ensure that even if we only used trials with similar motor behavior during R0 and R1 trials from the MC block, the neural activity would still show significant reward-related differences. Note NHP P didn’t show any differences between behavioral parameters under study. We pruned trials from the MC data block (NHP S) that had higher force duration (>2s) and reaction times (>1.5s), such that the remaining trials showed no difference between R0 and R1 trials. See Fig.S20 figure legend for the results of this comparison.
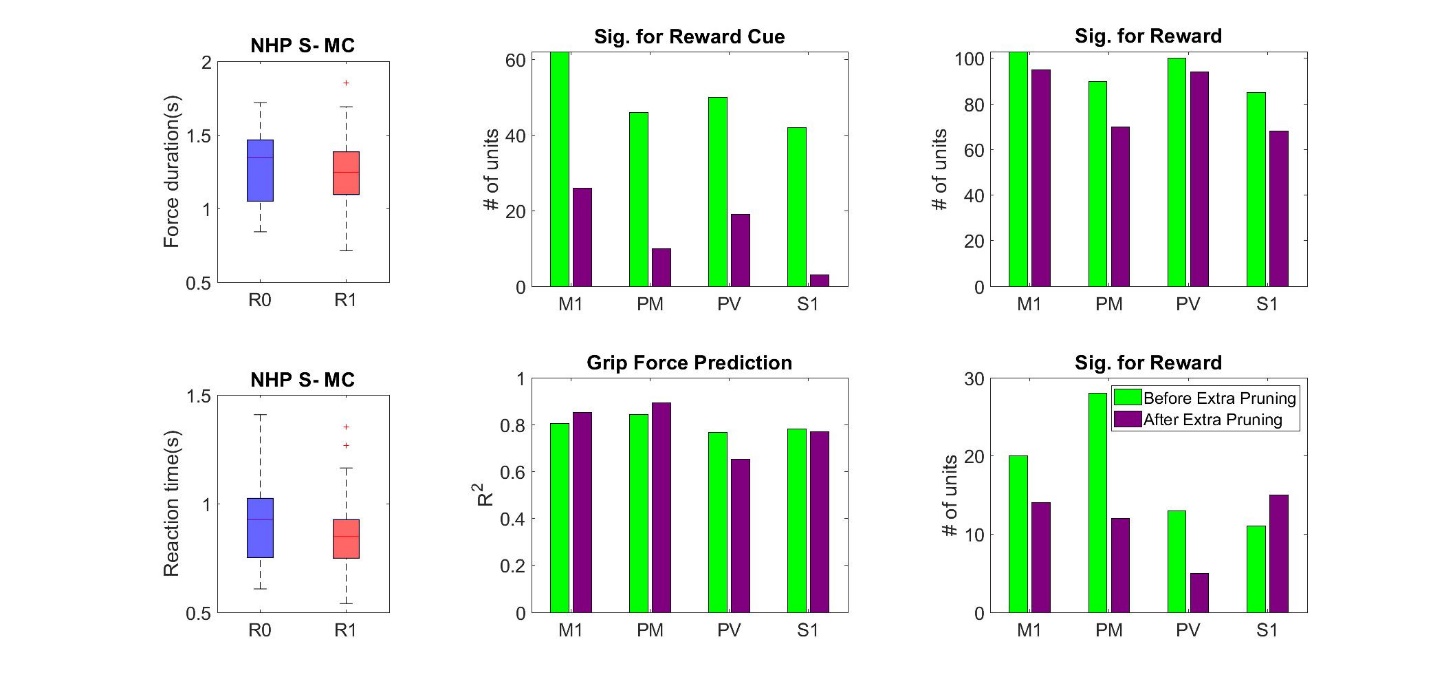
 **Figure S20:** Statistically significant results from the MC block for NHP S before and after extra pruning of the trials with non-significantly different motor behavior. The mean force duration and reaction time for R0 and R1 trials are shown in the left column, respectively. The number of significant units for reward cue, reward grip-force prediction.

**Mean Spike rate for observational Data blocks:**Figures 3 (NHPS) and 4 (NHPP) in the main text show raster plots and mean spike rates for example units from S1, M1, PMd, and PMv. For some cases (e.g., Fig 3, b1-c1), the variation due to force onset or offset is hard to observe from observational blocks, as the manual blocks had a stronger response compared to observational tasks, and in the main text figures, we used the same y-axis limits for both manual and observational cases. Figures S21 (NHP S) and S22 (NHP P) show the mean spike rate for observational blocks (OC and OU) for the example units already shown in Figures 3 and 4 in the main text. All these units showed significant fits (F-test, FDR corrected for p=0.05) for grip-force during manual and observational data blocks.


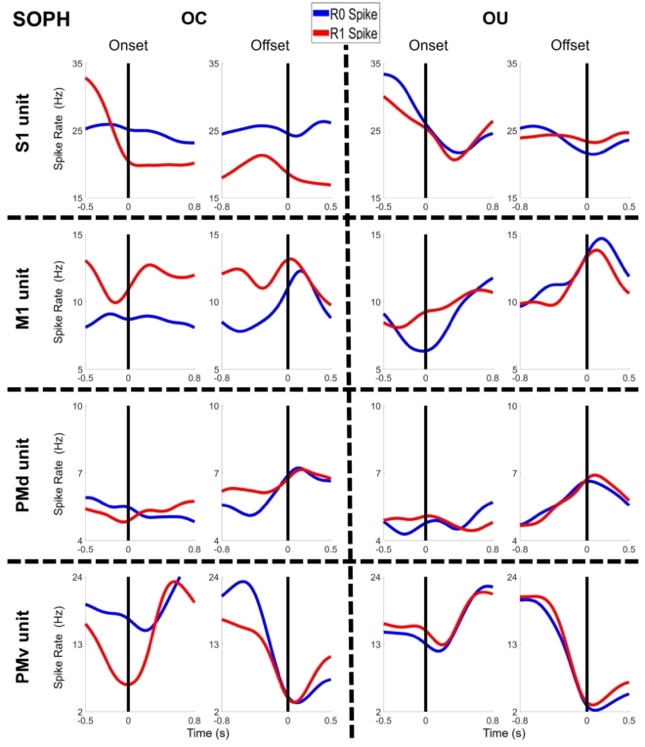


**Figure S21:** Mean spike rate for example units (fig 3) from NHP S. Only observational blocks are shown here.


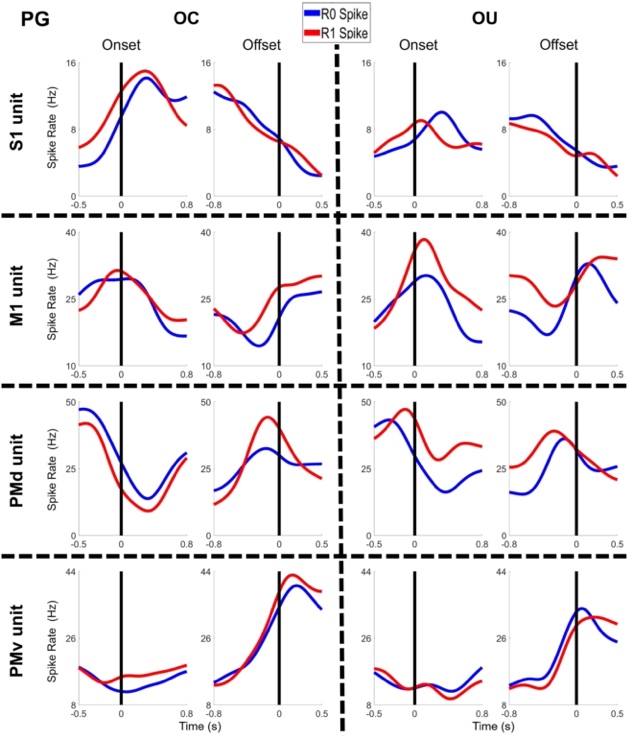


**Figure S22:** Mean spike rate for example units (fig 3) from NHP P. Only observational blocks are shown here.

1. [↑](#footnote-ref-1)
